# Discovery of Pyrazole-Based Positron Emission Tomography Agent that Maps Histone Deacetylase 6 (HDAC6) in the Nonhuman Primate Brain

**DOI:** 10.1021/acs.jmedchem.5c02216

**Published:** 2025-10-21

**Authors:** Tomoteru Yamasaki, Norio Ohyabu, Takeshi Wakabayashi, Ignacio Ibáñez, Kouichi Iwanaga, Satoshi Yamamoto, Masahiko Hattori, Taku Sugita, Michiko Terada, Tomohiro Onishi, Sho Sato, Yohei Kosugi, Akihiro Takano, Paul McQuade, Takamitsu Maru, Naomi Inui, Masayuki Fujinaga, Wakana Mori, Yuji Nagai, Chie Seki, Shoko Uchida, Takafumi Minamimoto, Makoto Higuchi, Makoto Fushimi, Ming-Rong Zhang

**Affiliations:** † National Institute for Quantum Science and Technology, Inage-ku, Chiba 263-8555, Japan; ‡ Takeda Pharmaceutical Company Limited, Fujisawa, Kanagawa 251-8555, Japan; § Takeda Pharmaceutical Company Limited, Chuo-ku, Osaka 540-8645, Japan; ∥ Takeda Development Center Americas, Inc., Cambridge, Massachusetts 02142, United States; ⊥ 561471 Axcelead Drug Discovery Partners, Inc., Fujisawa, Kanagawa 251-0012, Japan

## Abstract

Histone deacetylase 6 (HDAC6) is a crucial target for the development of pharmaceuticals used in the treatment of neurodegenerative disorders. Here, we identified **16a** as a candidate of positron emission tomography (PET) tracer for HDAC6 imaging from pyrazole derivatives, which showed strong HDAC6 affinity (*K*_d_ = 1.66 nM) and higher accumulation in the brain of wild-type mice than in HDAC6 knockout mice. Following radiolabeling with fluorine-18, PET with [^18^F]**16a** exhibited heterogeneous uptake of radioactivity, corresponding to the biological distribution of HDAC6 in the monkey brain. These radioactive distributions were homogeneously diminished by the preadministration of ACY-775, a potent inhibitor of HDAC6, suggesting that radioactive accumulation in PET images could reflect the specific binding of [^18^F]**16a** with HDAC6. Thus, [^18^F]**16a** is a promising PET tracer for HDAC6 imaging that motivates future clinical research.

## Graphical abstract

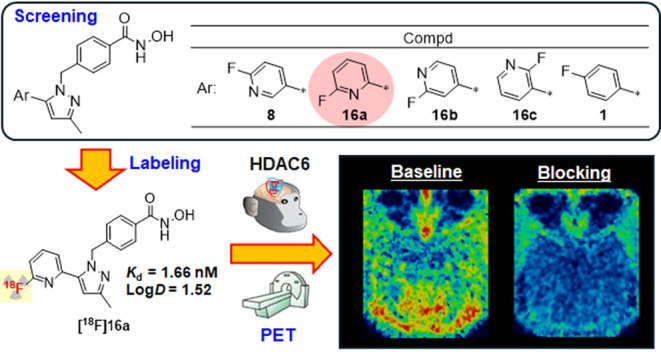

## Introduction

Histone deacetylase 6 (HDAC6) is a cytosolic enzyme that catalyzes the deacetylation of microtubules in axons. Inhibition of HDAC6 could improve axonal dysfunction through an increase in acetylated microtubules, thus leading to a potential treatment for neurodegenerative diseases with axonal damage, such as Alzheimer’s disease and Huntington’s disease.^1^ In a translational study of HDAC6 inhibitors targeting brain diseases, quantitative positron emission tomography (PET) imaging can be useful for estimating the target engagement of test drugs. In this context, several HDAC6 PET tracers have been developed, such as [^18^F]­bavarostat (also known as [^18^F]­EKZ-01),^2^ [^18^F]­FSW-100,^3^ and [^18^F]­PB118 (Figure 1).^4^ Of these, [^18^F]­bavarostat is a highly selective, brain-penetrant HDAC6 PET tracer that shows promise for imaging neuropsychiatric disorders. However, its clinical application may be limited by sex-based differences in tracer uptake, prolonged scan durations, and the need for further validation in patient populations.^5^ To explore more practical imaging agents for HDAC6, [^18^F]­FSW-100 and [^18^F]­PB118 have been developed as novel PET tracers based on the *N*-hydroxybenzamide scaffold. Although [^18^F]­FSW-100 demonstrated high specific uptake in HDAC6-rich brain regions, its brain kinetic profile complicates quantitative PET analysis.^3^ In contrast, [^18^F]­PB118 exhibited favorable brain kinetics, specificity, and selectivity for HDAC6. Nevertheless, its radiosyhthesis involved two high-performance liquid chromatography (HPLC) purification steps, which pose challenges for automation using standard radiosynthesizers.^4^ These limitations highlight the ongoing need for the development of novel HDAC6 PET tracers that are both synthetically accessible and suitable for quantitative brain imaging.

**Figure 1:**
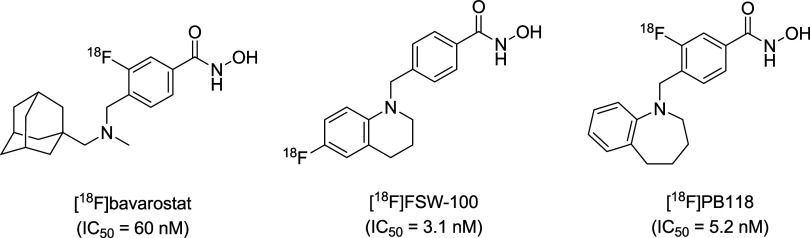
Representative HDAC6 PET tracers with IC_50_ values for HDAC6.

Recently, pyrazole derivatives represented by compound 11i (**1**) were reported as a new class of HDAC6 inhibitors by Shenyang Pharmaceutical University.^6^ This compound has a common zinc-binding benzohydroxamic acid attached to a characteristic phenyl pyrazole moiety that would be accommodated in the capping region of the enzyme to exert isoform selectivity and potency. From a PET perspective, this class is attractive because of a high central nervous system (CNS) multiparameter optimization score with relatively low lipophilicity, implying sufficient brain penetration and low nonspecific binding in the PET imaging.^7^ In addition, the pendant phenyl group could be replaced with a 2-fluoropyridine moiety, which may result in lower nonspecific binding and higher labeling accessibility, while maintaining brain penetration. (Figure 2).^8^

**Figure 2:**
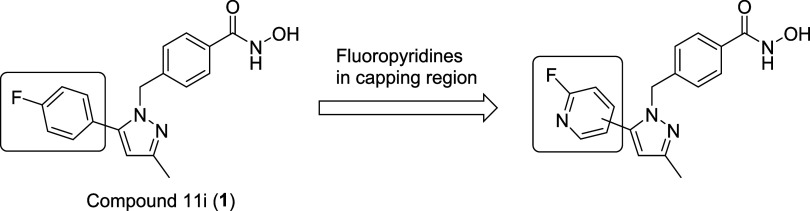
Compound 11i (**1**) and 2-fluoropyridine derivative.

In this study, we explored the installation of 2-fluoropyridine as a substitute for the 4-fluorophenyl group of compound **1**, with the aim of discovering a new class of brain-penetrant HDAC6 PET tracers. Compound screening was performed based on the evaluation of HDAC6 inhibitory activity and isoform selectivity, as well as multidrug resistance protein 1 (MDR1) substrate liability, which resulted in the identification of **16a** as a potent and blood–brain barrier-permeable HDAC6 inhibitor. Compound **16a** showed strong HDAC6 affinity in an *in vitro* binding assay and showed higher accumulation in the brains of wild-type (WT) mice than in HDAC6 knockout (KO) mice after intravenous administration. Compound **16a** was successfully ^18^F-labeled with high radiochemical yield using a conventional protocol. In a nonhuman primate (NHP) PET baseline study, [^18^F]**16a** showed rapid clearance and regional heterogeneous distribution with a peak standardized uptake value (SUV) of approximately 1. Moreover, administration of ACY-775,^9^ a known HDAC6 inhibitor, completely blocked the retention of [^18^F]**16a** in the NHP brain. Herein, we report the discovery of **16a** and the results of NHP-PET imaging using [^18^F]**16a**.

## Results and Discussion

### Chemistry

Fluoropyridine derivatives **8** and **16a**–**c** were synthesized according to Schemes 1 and 2, respectively. Benzylation of pyrazole **2** with **3** yielded **4** as a minor product together with its isomer, which was separated using column chromatography. The structure of **4** was determined using X-ray diffraction. Compound **4** was converted to **5** via hydrolysis, followed by amidation with *O*-THP protected hydroxylamine, Suzuki–Miyaura coupling with (6-fluoropyridin-3-yl)­boronic acid, and acidic deprotection of the THP group to provide **8** (Scheme 1).

**Scheme 1:**
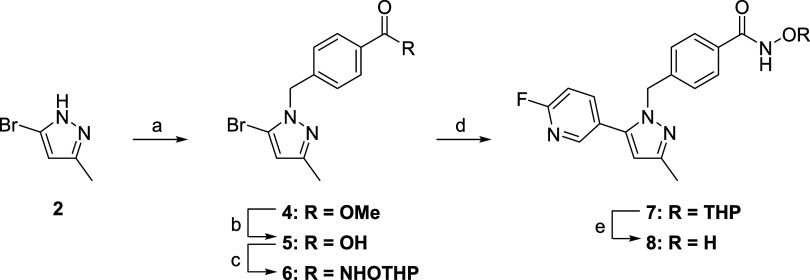
Synthesis of Compound **8** ^a^

a Reagents and conditions: (a) methyl 4-(bromomethyl)­benzoate (**3**), Cs_2_CO_3_, CH_3_CN, rt, 25%; (b) 1 M NaOH, THF, MeOH, rt, 98%; (c) H_2_N-OTHP, EDC·HCl, 1-hydroxybenzotriazole (HOBt), triethylamine (TEA), DMF, rt, 93%; (d) (6-fluoropyridin-3-yl)­boronic acid, PdCl_2_(Amphos)_2_, K_2_CO_3_, DME, water, 100 °C; (e) 0.1 M HCl, THF, MeOH, 0 °C–rt, 51% for 2 steps.

**Scheme 2:**
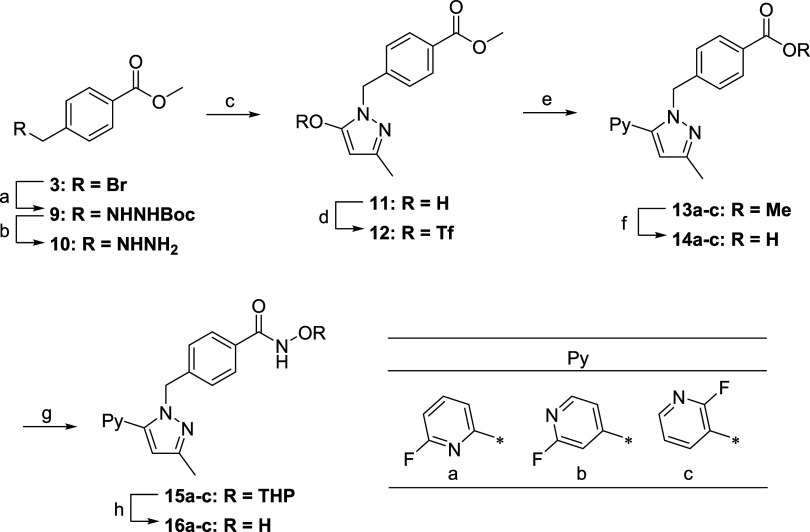
Synthesis of Compound **16a**–**c** ^a^

a Reagents and conditions: (a) *tert*-butyl carbazate, TEA, DMF, rt–75 °C, 74%; (b) 4 M HCl in dioxane, 0 °C–rt; (c) methyl 3-oxobutanoate, acetic acid, 125 °C, 30% for 2 steps; (d) PhNTf_2_, *N,N*-diisopropylethylamine (DIPEA), DCM, 40 °C, 64%; (e) 2-(6-fluoropyridine)­cyclic-triolborate lithium salt, Pd­(Amphos)_2_Cl_2_, CuCl, K_3_PO_4_, DME, water, 100 °C, microwave irradiation for **13a**, 59%; PyB­(OH)_2_, 1,1′-bis­(diphenylphosphino)­ferrocene (dppf), PdCl_2_(dppf), K_3_PO_4_, dioxane, 100 °C for **13b** and **13c**, 90–97%; (f) TEA, MeOH, water, 70 °C for **14a**, 94%; KOSiMe_3_, THF, 0 °C–rt for **14b** and **14c**, 84–91%; (g) H_2_N-OTHP, EDC·HCl, HOBt, TEA, DMF, rt; (h) 0.1 M HCl, MeOH–THF (1/1), rt, 18–54% for 2 steps.

Due to the undesirable selectivity of *N*-benzylation of **2** in the above route, an alternative method was developed to prepare **16a**–**c** (Scheme 2). The reaction of **3** with *tert*-butyl carbazate, followed by deprotection of the Boc group, produced hydrazine **10**, which was cyclized with methyl 3-oxobutanoate to form hydroxypyrazole **11**. After *O*-triflation, three different pyridyl groups were introduced using Suzuki–Miyaura coupling to yield **13a**–**c**. Finally, the ester group of **13a**–**c** was converted to hydroxamic acid in a manner similar to that of the former route to furnish **16a**–**c**.

### Exploration

The four synthesized fluoropyridine derivatives were evaluated for their HDAC-inhibitory activity, MDR1 susceptibility, free fraction with a mouse brain homogenate, and Log *D* (Table 1). All the compounds showed HDAC6 inhibitory activity comparable to that of the parent compound (**1**) with nanomolar potency and little species difference. Although the HDAC6 selectivity of the four compounds was generally high, **16a** and **16b** showed superior selectivity compared to **8** and **16c**, especially over HDAC1. MDR1 susceptibility was dependent on the Log *D* value;^10^ the relatively less-hydrophobic compounds **8** and **16c** were likely to be MDR1 substrates, as indicated by an MDR1 efflux ratio >2. Due to low lipophilicity with Log *D* values <2, these compounds displayed a high free fraction rate >10%, which portends a low nonspecific binding signal in the PET imaging.^11^ Overall, **16a** showed the best profile with a nanomolar potency, little MDR1 substrate liability, and high free fraction, and thus moved forward to further assessment toward the development of the HDAC6 PET tracer.

**Table 1:**
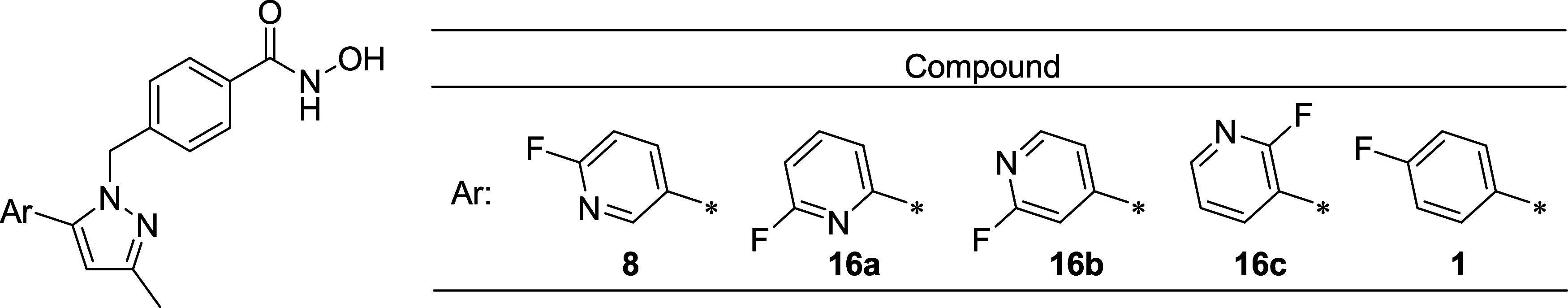
Inhibitory Activity of Histone Deacetylase 6 (HDAC6), MDR1 Susceptibility, Mouse *F*_ub_, and Log *D*

	IC_50_ (nM)^a^						MDR1^b^			
Compound	hHDAC6	mHDAC6	hHDAC1	hHDAC4	hHDAC7	hHDAC8	Papp A-to-B	Ratio	*F*_ub_ ^c^ (%)	Log *D*
**8**	5.2 (3.1–8.7)	8.7 (8.0–9.5)	350 (300–420)	>10000	2300 (1600–3300)	260 (220–300)	79	2.3	23	0.98
**16a**	2.7 (2.4–3.1)	3.7 (3.5–4.0)	1300 (1100–1600)	>10000	4400 (3500–5500)	640 (590–690)	94	1.2	12	1.52
**16b**	5.6 (4.9–6.5)	NT	1300 (1100–1500)	>10000	4000 (2600–6300)	520 (490–550)	122	1.7	22	1.00
**16c**	7.4 (6.1–9.0)	NT	590 (530–670)	>10000	4900 (4000–6000)	NT	31	3.3	NT	0.89
**1**	4.3 (3.6–5.1)	3.4 (3.0–3.7)	430 (390–470)	>10000	810 (630–1000)	530 (490–580)	109	0.96	3.3	2.04

a The activity was evaluated with an enzymatic HDAC-Glo I/II assay after 1 h preincubation of the test compounds with the human or mouse enzymes. IC_50_ values are shown with 95% confidence intervals given in parentheses (*n* = 2).

b Absorptive apparent permeability coefficient (Papp A-to-B, nm/sec) and efflux ratio in MDR1-overexpressing cells at 1 μM substrate are exhibited.

c The free fraction in mouse brain homogenates. NT, not tested.

### Affinity Selection (AS)–Mass Spectrometry (MS) Binding Study

We next assessed the binding of **16a** to HDAC6 using an affinity selection (AS)–MS technique, together with bavarostat, the sole HDAC6 PET tracer tested clinically in the time period of this study.^12^ The results are summarized in Figure 3. In the direct binding assay, **16a** and bavarostat showed a saturable binding to HDAC6 with dissociation constants (*K*_d_) of 1.66 and 11.7 nM, respectively, which considered to be consistent with the IC_50_ values obtained in the functional assay. The dissociation assay indicated that *T*_1/2_ of **16a** and bavarostat were 15.9 and 8.87 min, respectively. Using these parameters, *k*_off_ and *k*_on_ were calculated as 7.27 × 10^–4^ s^–1^ and 4.38 × 10^5^ M^–1^ s^–1^ for **16a**, respectively, and 1.30 × 10^–3^ s^–1^ and 1.11 × 10^5^ M^–1^ s^–1^ for bavarostat, respectively. These intrinsic kinetic parameters directly demonstrate the greater HDAC6 binding affinity of **16a** relative to bavarostat and warrant further studies on **16a** as an alternative HDAC6 tracer.

**Figure 3:**
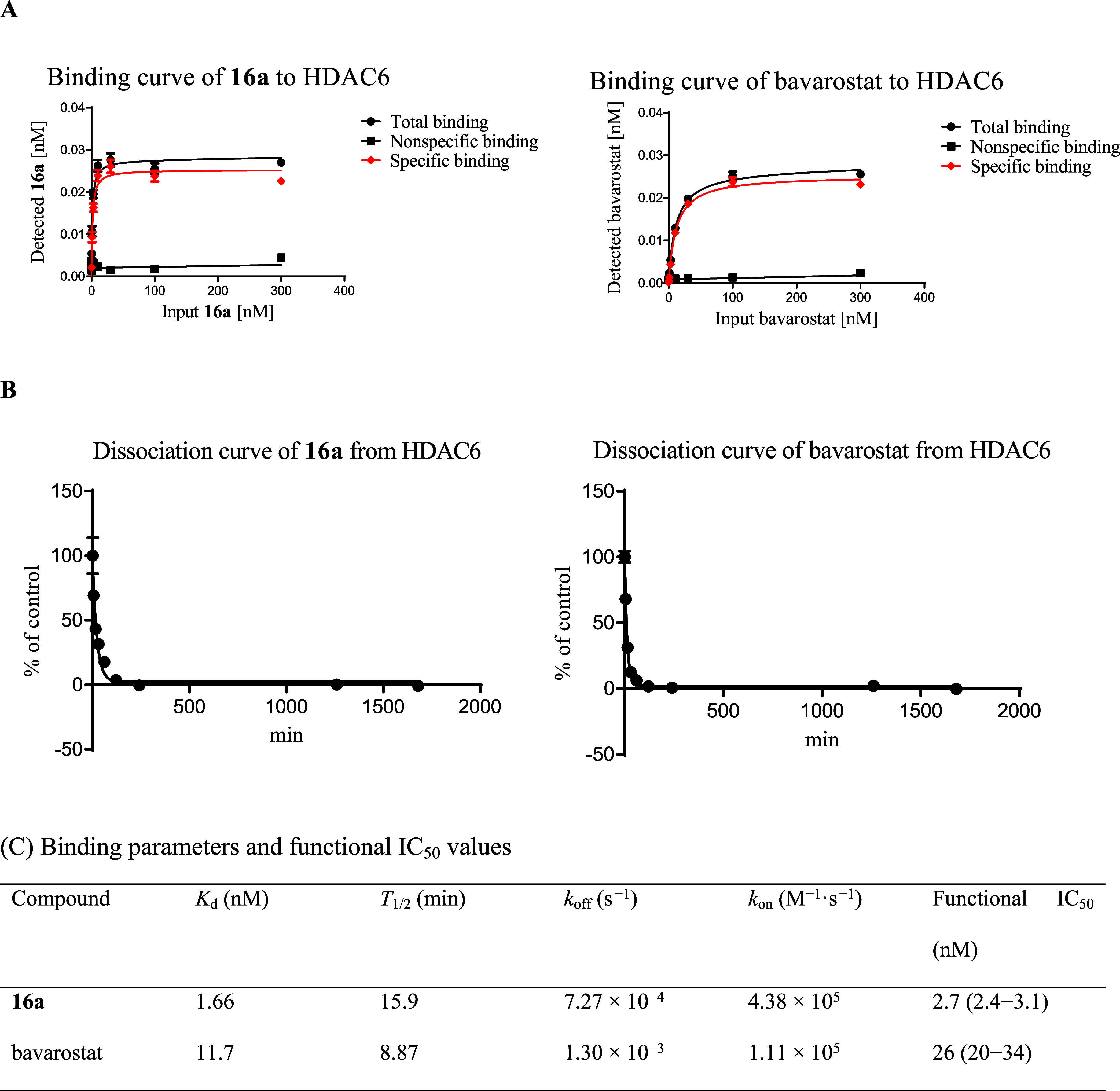
Affinity selection (AS)–MS binding study of HDAC6 with **16a** and bavarostat. Data points are the mean ± standard error (SE) (four technical replicates). (A) Saturation binding curve. (B) Time course for the dissociation. (C) Binding parameters calculated from AS–MS data. Functional IC_50_ values are shown for comparison.

### Mouse Brain Uptake Study

Next, we examined the brain accumulation of **16a** in wild-type (WT) and HDAC6 knockout (KO) mice.^13^ Compound **16a** was intravenously administered to WT and HDAC6 KO mice at a dose of 1 mg/kg each; 2 h after administration, the amount of **16a** in the hippocampus, the HDAC6-rich region,^2,4,5^ and plasma was measured using liquid chromatography with tandem mass spectrometry (LC–MS/MS). The brain concentration of **16a** in WT mice was 2-fold higher than that in HDAC6 KO mice, with comparable plasma concentrations (Figure 4). These results indicate that **16a** is retained in the brain in a manner related to HDAC6 expression.

**Figure 4:**
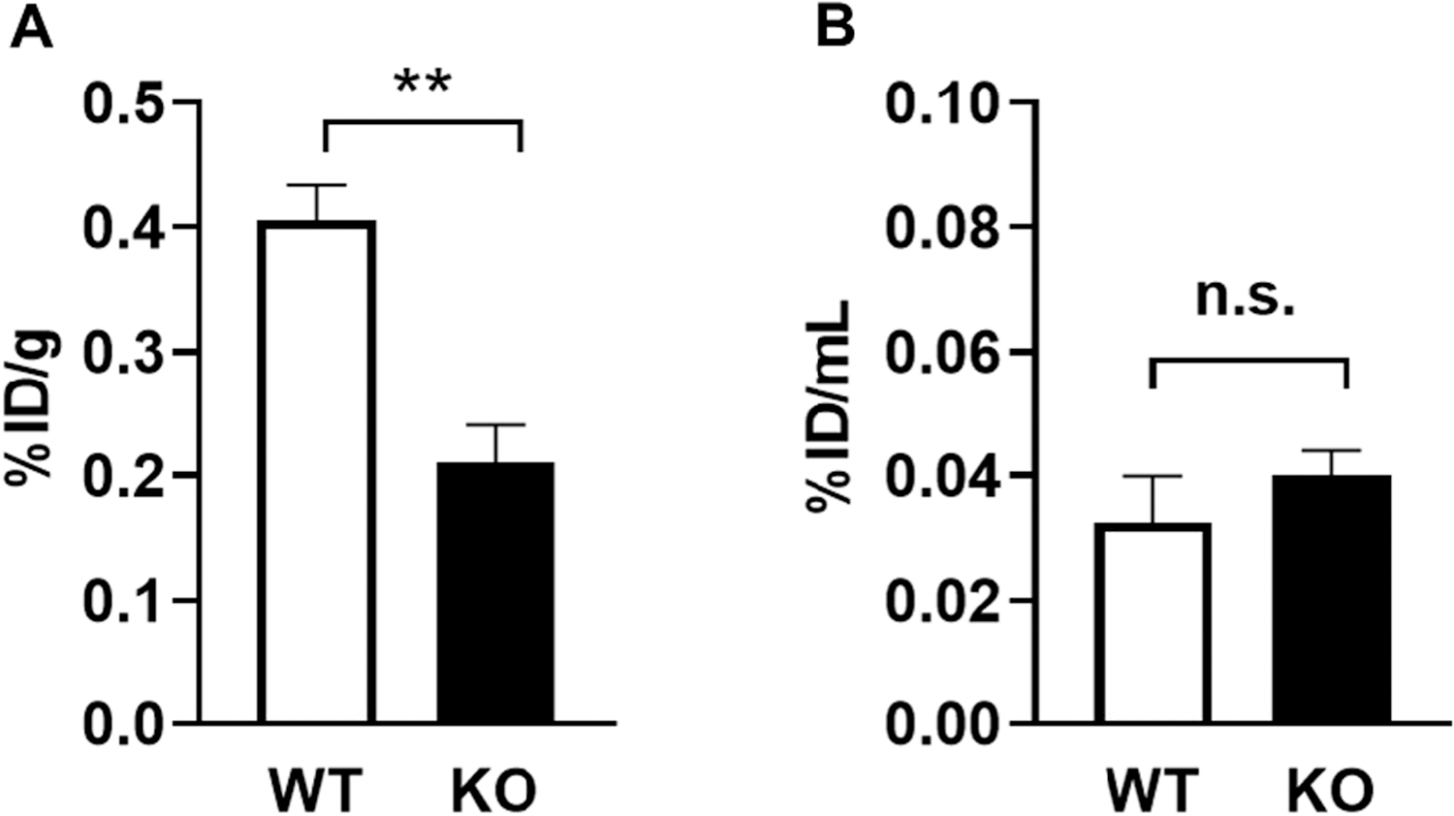
Brain and plasma concentration of **16a** in wild-type (WT) and HDAC6 knockout (KO) mice. Compound **16a** (1 mg/kg) was intravenously administered to WT and HDAC6 KO mice. Two hours after administration, the hippocampus and plasma were collected and the concentration of **16a** in the brain (A) and plasma (B) was measured using liquid chromatography with tandem mass spectrometry (LC–MS/MS). The data are expressed in percentage of injected dose per gram of brain tissue (%ID/g) or per milliliter of plasma (%ID/mL), respectively, as mean ± standard error (SE), *n* = 4. **P < 0.01, n.s.: not significant.

### HDAC6 Expression Level in Mouse and Monkey Brain

Toward testing **16a** in a binding study with monkeys, we first examined the expression level and distribution of the HDAC6 protein in the brain of monkeys. The amount of HDAC6 in the hippocampus and corpus callosum of monkeys was assessed using Western blotting and compared with that in the mouse hippocampus (Figure 5). For semiquantitative analysis, 0.03, 0.1, 0.3, and 1 ng of human recombinant HDAC6 were evaluated in each experiment, and comparable HDAC6 expression levels were observed in the hippocampi of mice and monkeys.

**Figure 5:**
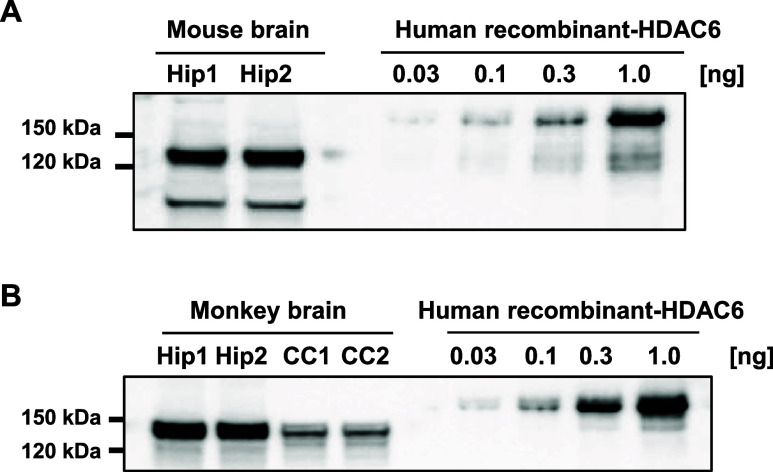
Comparison of HDAC6 expression levels in a mouse and monkey using Western blotting. Hippocampus (Hip) was obtained from two mice, and HDAC6 expression was examined. Recombinant HDAC6 conjugated with GST (0.03, 0.1, 0.3, and 1 ng) was also used for the positive controls (A). Lysates of the hippocampus and corpus callosum (CC) obtained from a monkey were applied to Western blotting in duplicate. The same controls were used for comparison (B).

The expression level was lower in the corpus callosum of monkeys, suggesting that HDAC6 expression was higher in the gray matter than in the white matter. This tendency was confirmed by the immunohistochemical (IHC) study. Coronal sections of the monkey brain were prepared and immunostained with an HDAC6 antibody (Figure 6). In accordance with the results of Western blotting, HDAC6 expression was high in the gray matter of the putamen, cerebral cortex, hippocampus, and cerebellum, whereas its expression in the corpus callosum and pons was relatively low.

**Figure 6:**
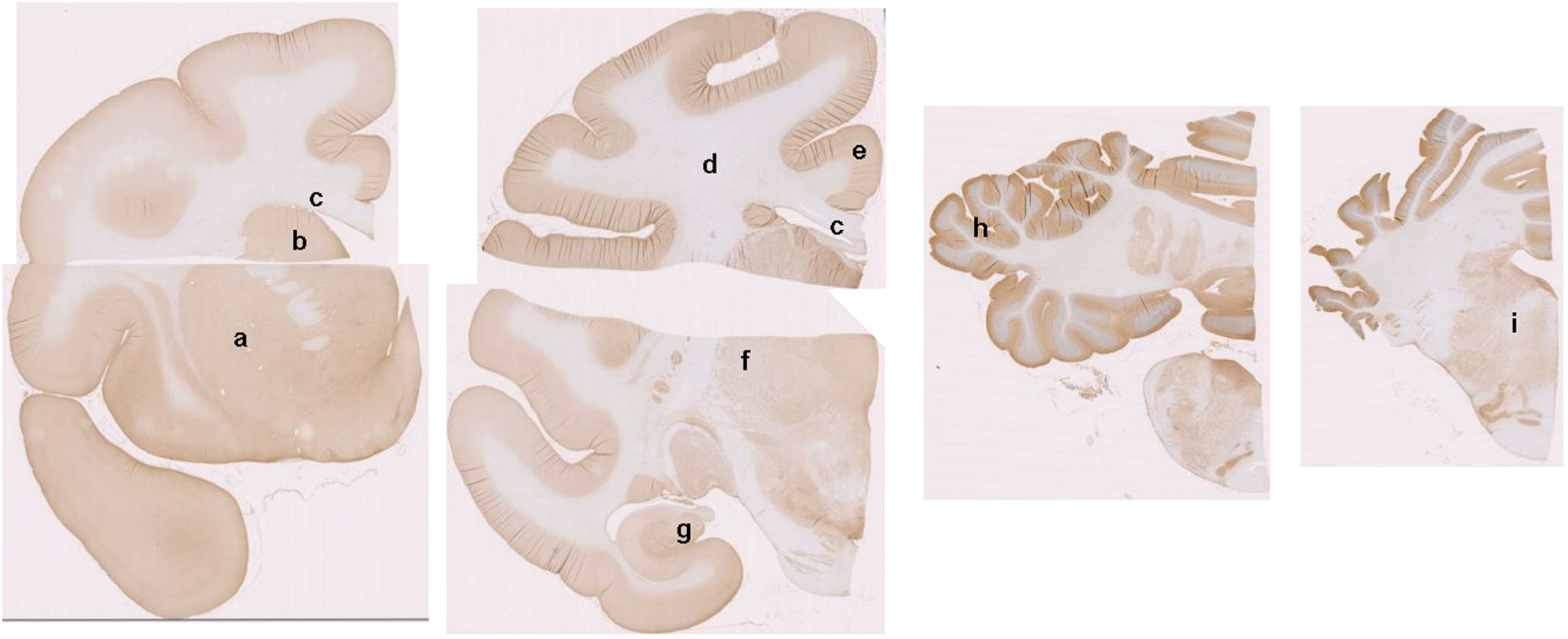
Immunohistochemical (IHC) staining of HDAC6 protein in a monkey brain. Coronal brain sections, including the areas below were prepared from a monkey (a, putamen; b, caudate nucleus; c, corpus callosum; d, white matter; e, anterior cingulate cortex; f, thalamus; g, hippocampus; h, cerebellum; i, brain stem [pons]). They were immunostained with HDAC6 antibody.

### Radiolabeling

According to the basic strategy mentioned earlier, 2-nitropyridine **19** was designed as a precursor for [^18^F]**16a** and synthesized based on one-pot Suzuki biaryl synthesis.^14^ At first, compound **6** was subjected to one-pot iterative Suzuki–Miyaura coupling with bis­(pinacolato)­diboron followed by 2-chloro-6-nitropyridine; however, a poor yield of the desired coupling product was obtained (data not shown), unlike the reactions shown in Scheme 2. Although we did not determine the reason for this observation, we found that it was useful to change the starting material to ester **4** without a hydroxamate moiety. Compound **4** was converted to the boronic acid ester and subsequently coupled with 2-chloro-6-nitropyridine under Suzuki–Miyaura conditions to produce compound **17**, which was transformed to compound **19** via carboxylic acid **18** in a manner similar to that described in previous schemes (Scheme 3).

**Scheme 3:**
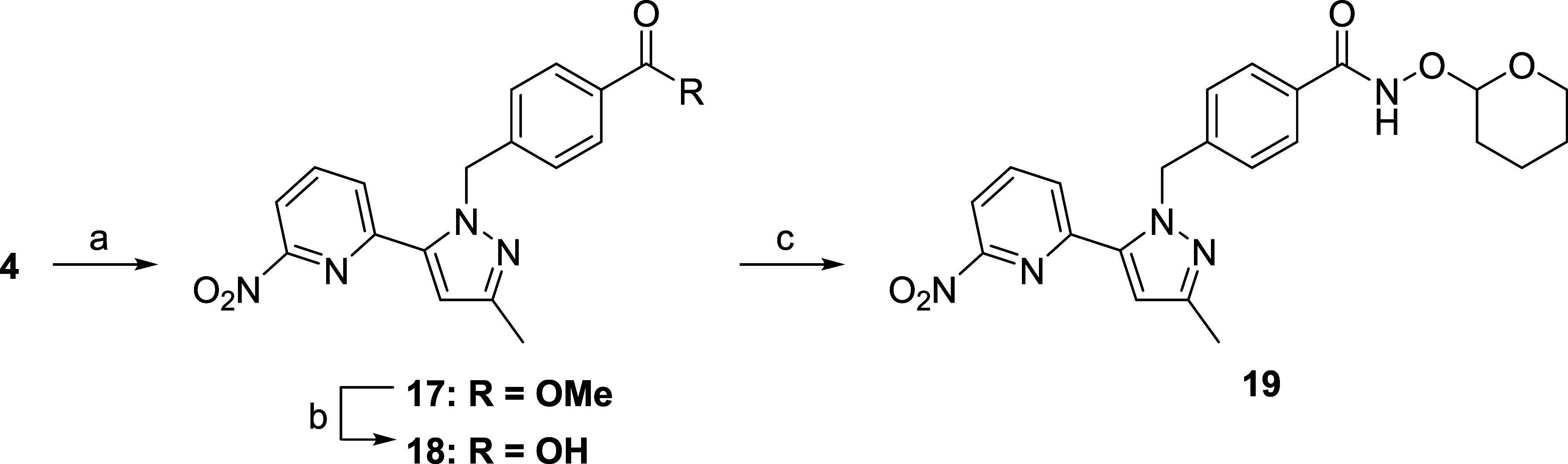
Synthesis of Precursor **19** ^a^

a Reagents and conditions: (a) (i) bis­(pinacolato)­diboron, PdCl_2_(PCy_3_)_2_, KOAc, cyclopentyl methyl ether, 100 °C; (ii) 2-chloro-6-nitropyridine, PdCl_2_(PCy_3_)_2_, 1 M K_2_CO_3_, cyclopentyl methyl ether, 100 °C, 85%; (b) 1 M NaOH, THF, water, 80 °C, 89%; (c) H_2_N-OTHP, EDC·HCl, HOBt·H_2_O, DIPEA, DMF, 0 °C–rt, 47%.

Radiosynthesis of [^18^F]**16a** was prepared according to Scheme 4 using a homemade automated synthesis system.^15^ A mixture of the 2-nitropyridine precursor **19** and dried [^18^F]­fluoride in DMSO was heated at 150 °C for 10 min, followed by adding 1 M HCl into the reaction mixture (Scheme 4). An amount of 1.7–2.3 GBq of [^18^F]**16a** was produced from 8.9 GBq of [^18^F]­fluoride over a period of 86 ± 1 min (*n* = 3) synthesis time from end of bombardment (EOB). The radiochemical yield of [^18^F]**16a** was 38 ± 5% (*n* = 3, decay-corrected) based on [^18^F]­fluoride. The specific activity of [^18^F]**16a** was 356 ± 74 GBq/μmol (*n* = 3). The identity of the product was confirmed using HPLC by coinjecting unlabeled **16a** (Figure S2).

**Scheme 4:**
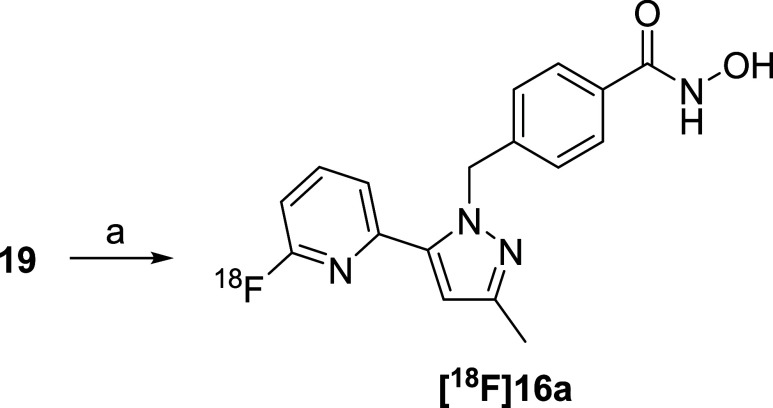
Synthesis of [^18^F]**16a** ^a^

a Reagents and conditions: (a) (i) [^18^F] KF/kryptofix 222., DMSO, 150 °C, 10 min; (ii) HCl, 90 °C, 4 min.

### *In Vitro* Autoradiography in Rat Brain

Prior to PET imaging, *in vitro* autoradiography with [^18^F]**16a** using rat brain sections was performed to evaluate specific binding of [^18^F]**16a** with HDAC6. Figure 7A and B show representative autoradiographs of [^18^F]**16a** with or without self-blocking (1 μM), using sagittal brain sections of rats. In the control section, radioactivity was heterogeneously distributed across brain regions in the following order: striatum > cerebral cortex ≈ hippocampus > cerebellum > thalamus > pons. This regional radioactive accumulation was homogeneously diminished with significant differences by coincubation with 1 μM of unlabeled **16a**, indicating effective self-blocking. These results demonstrated a heterogeneous distribution of [^18^F]**16a** that corresponded well with the known biological distribution of HDAC6.^2,4,5^ Furthermore, when compared to [^18^F]­bavarostat,^2^ [^18^F]**16a** exhibited superior specific binding, suggesting its potential as a more effective imaging agent for HDAC6. This enhanced binding profile may contribute to improved visualization of HDAC6-rich brain regions in PET studies.

**Figure 7:**
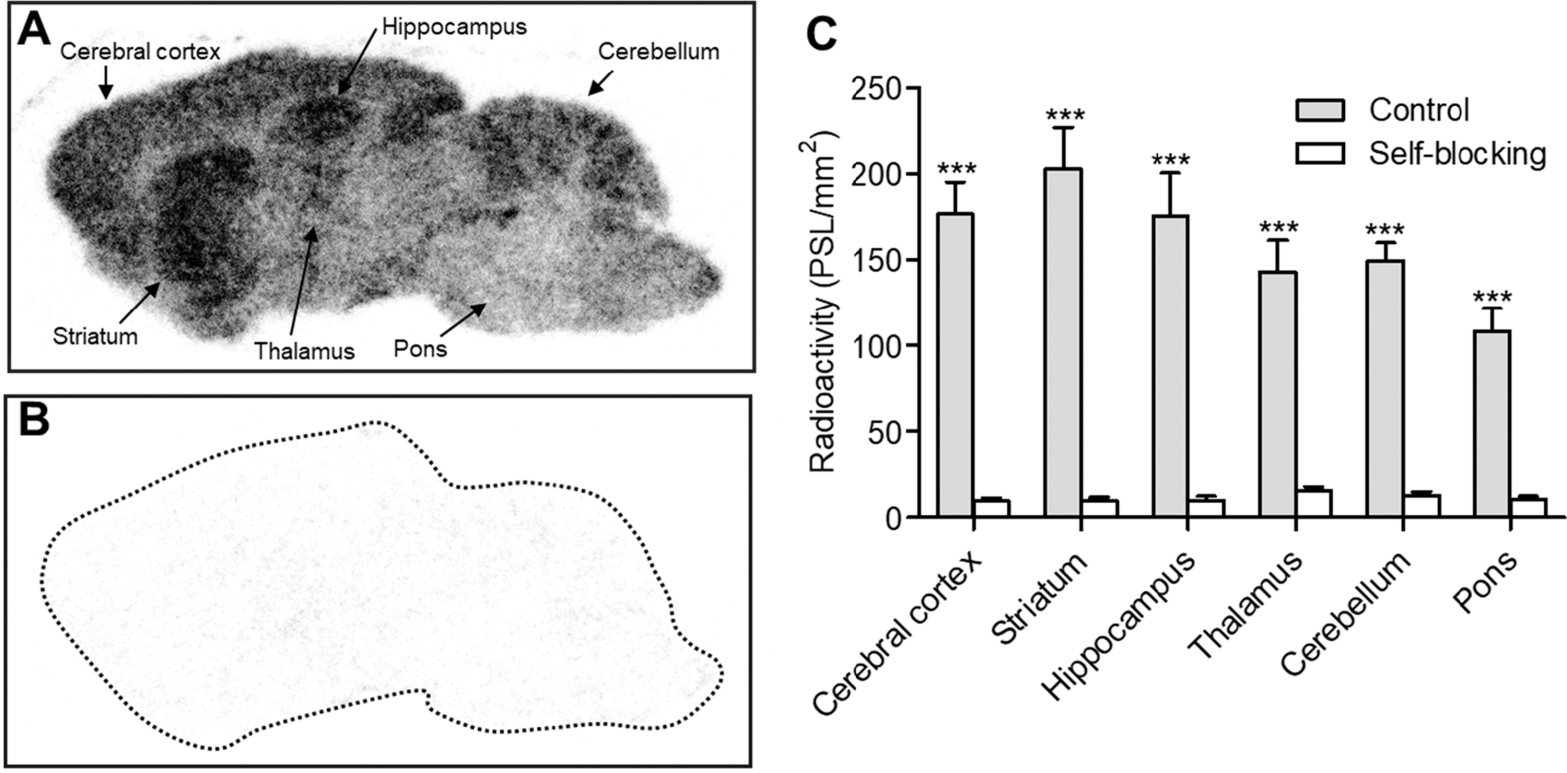
*In vitro* autoradiography with [^18^F]**16a** using rat brain sections. Representative autoradiograms were obtained by incubating sagittal brain sections with [^18^F]**16a** solution including either DMSO (vehicle) (A) or unlabeled **16a** (1 μM) (B). Regions of interest were drawn on the cerebral cortex, striatum, hippocampus, thalamus, cerebellum, and pons. Radioactivity is expressed as photostimulated luminescence (PSL)/mm^2^. ***P < 0.001 (control vs self-blocking).

### NHP PET Imaging

The *in vivo* performances of [^18^F]**16a** were evaluated using PET scans with blood sampling using a rhesus monkey. Both plasma input curves of [^18^F]**16a** in the baseline and blocking (2 mg/kg of ACY-775) studies contained approximately 60% of unchanged form 5 min after the injection and then decreased to <20% at 15 min and ∼10% at 30 min, which finally reached ∼5% at the end of the PET scan (Figure S3). Although plasma metabolite analysis revealed three polar radiolabeled metabolites (Figure S4), their low lipophilicity suggests that they are unlikely to cross the blood–brain barrier. Figure 8A and B show time–activity curves (TACs) of [^18^F]**16a** in the brain of rhesus monkeys treated with or without ACY-775 (2 mg/kg), a potent inhibitor of HDAC6. Representative regions of interest (ROIs) for TAC acquisition were drawn in the dorsolateral frontal cortex (DFC), white matter, putamen, hippocampus, cerebellum, brainstem, and corpus callosum. The highest radioactive uptake with a peak of 0.9 SUV in the baseline PET scan was observed in the cerebellum, followed by moderate radioactive uptakes (peak of 0.6–0.8 SUV) ranked as DFC, putamen, hippocampus, and white matter. Low radioactive uptake (peak <0.6 SUV) was observed in the brain-stem and the corpus callosum. IHC staining for HDAC6 was negligible not only in the brainstem and corpus callosum, but also in the white matter (Figure 6). The radioactive uptake in the white matter would be due to some radioactivity spilling over from the gray matter of the cortex. Overall, the order of radioactive uptake closely corresponded to the IHC results for HDAC6 (Figure 6), with low radioactive accumulation in the skull due to defluorination of [^18^F]**16a**. Moreover, by pretreatment with ACY-775, radioactive accumulations in all regions decreased substantially to ∼0.3 SUV without regional differences. These results strongly suggest that [^18^F]**16a** specifically binds to HDAC6 *in vivo*. Nevertheless, we currently have no data regarding the off-target binding profile of [^18^F]**16a**. Given that several HDAC6 inhibitors, including ACY-775, have been reported to bind off-target to the metallo-β-lactamase domain-containing protein 2,^16^ we cannot exclude the possibility that such interactions may contribute to the observed PET signal.

**Figure 8:**
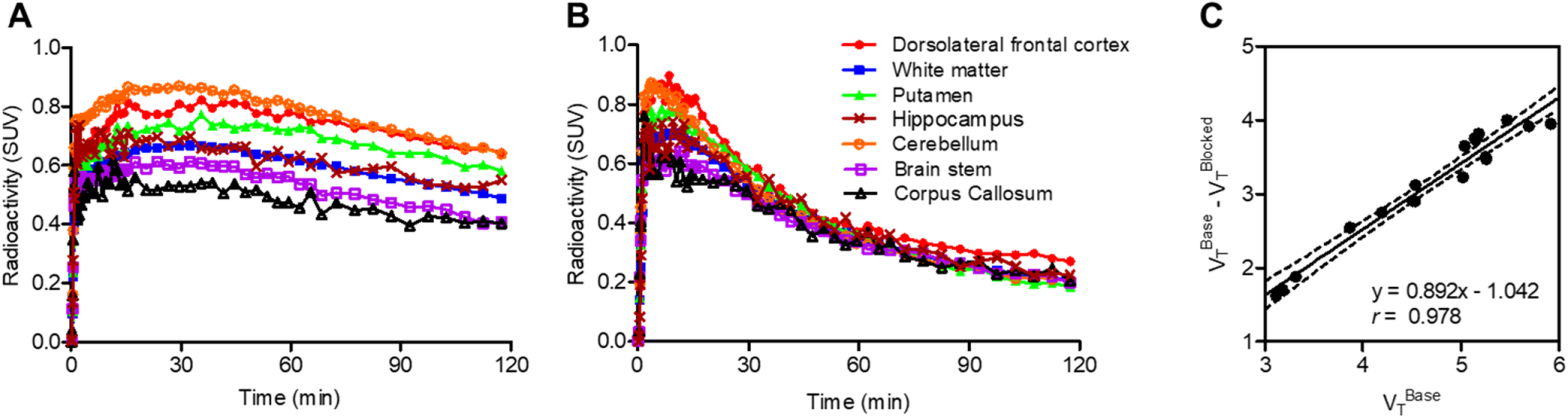
Time–activity curves (TACs) after an intravenous injection of [^18^F]**16a** in the brain of a rhesus monkey pretreated without (A) or with ACY-775 (2 mg/kg, 5 min before) (B) and Lassen plots (C) using distribution volumes in PET imaging with [^18^F]**16a**. The TACs in representative regions of interest were obtained in the dorsolateral frontal cortex, white matter, putamen, hippocampus, cerebellum, brain stem (containing midbrain, pons, and medulla), and corpus callosum with reference to the NIMH Macaque template. The radiotracer concentrations are expressed as standardized uptake volumes (SUVs).

Although the radioactive uptake of [^18^F]**16a** in all ROIs was lower than that of [^18^F]­FSW-100, brain kinetics with rapid clearance of radioactivity in PET with [^18^F]**16a** would be more reasonable for quantitative analysis than [^18^F]­FSW-100, which has a very slow clearance of radioactivity from the brain.^17^ Distribution volumes (*V*_T_s), widely used as the quantitative value for radioactive uptake, of [^18^F]**16a** in brain regions were estimated using the plasma input curve using Logan graphical analysis (Logan GA).^18^ The highest *V*_T_ value was determined in the DFC (5.8 mL/cm^3^), slightly exceeding that of the cerebellum (5.5 mL/cm^3^), as shown in Table 2. The *V*_T_-based parametric PET images of [^18^F]**16a** at baseline showed heterogeneous signals, with strong signals observed in the DFC, cerebellum, and gray matter of the cortex (Figure 9B). The corpus callosum exhibited the lowest signals. Upon treatment with ACY-775, the *V*_T_-based signals in the PET images decreased homogeneously (Figure 9C), suggesting that the baseline *V*_T_ value reflected the volume of specific binding of [^18^F]**16a** to HDAC6. Estimating using the Lassen plot (Figure 8C) indicated that treatment with ACY-775 occupied approximately 90% of the binding site of HDAC6, and the *V*_T_ for free or nonspecific binding of [^18^F]**16a** (defined as *V*_ND_) was 1.2 mL/cm^3^. Thus, it was successfully demonstrated that [^18^F]**16a** specifically bound to HDAC6 *in vivo* in the monkey brain.

**Figure 9:**
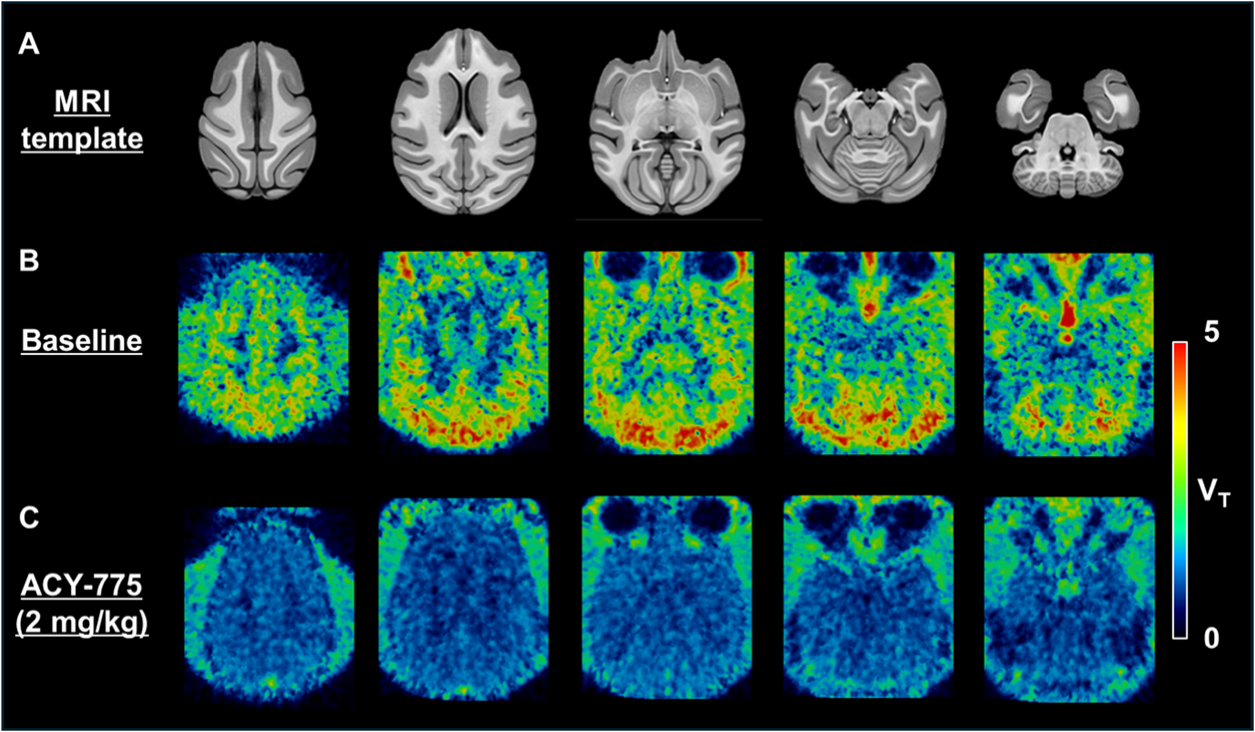
NIMH Macaque magnetic resonance imaging (MRI) template (A) and parametric positron emission tomography (PET) images scaled with distribution volume (*V*_T_) of [^18^F]**16a** in the brain of a rhesus monkey pretreated without (B) or with ACY-775 (2 mg/kg, 5 min before) (C).

**Table 2:** Distribution Volume (*V*_T_) in Brain Regions of a Rhesus Monkey Administered ACY-775 (2 mg/kg) or Not

	*V*_T_ (mL/cm^3^)		
Brain regions	Baseline	ACY-775	% decrease
Dorsolateral frontal cortex	5.8	1.9	68
White matter	4.2	1.4	66
Putamen	5.1	1.4	64
Hippocampus	4.5	1.5	66
Cerebellum	5.5	1.5	73
Brain stem	3.4	1.4	58
Corpus Callosum	3.3	1.4	57

## Conclusion

In the present study, we identified [^18^F]**16a** from the pyrazole derivatives of compound 11i (**1**) as a novel PET tracer for HDAC6 imaging. Of the pyrazole derivatives, compound **16a** showed the strongest affinity for human HDAC6 and the highest subtype selectivity. Prior to PET assessment, radiolabeling of **16a** was successfully performed using the reaction of 2-nitropyridine precursor (**19**) with [^18^F]­KF, obtaining 1.7 GBq of [^18^F]**16a** (radiochemical yield of 38%). PET studies with [^18^F]**16a** using rhesus monkeys showed high *V*_T_ values in the HDAC6-rich regions of the brain. Moreover, the specific binding of [^18^F]**16a** to HDAC6 *in vivo* was demonstrated by blockade using ACY-775, a potent inhibitor of HDAC6. Hence, [^18^F]**16a** is a promising PET tracer for HDAC6 imaging, which motivates further clinical investigations to elucidate the mechanisms involving HDAC6 in CNS disorders.

## Experimental Section

### General

The ^1^H NMR spectra were recorded using a Bruker Ultra Shield 300 (300 MHz) or 400 (400 MHz) spectrometer. Chemical shifts are given in δ values (ppm) using TMS as the internal standard. All *J* values are given in Hz. The following abbreviations are used: s, singlet; d, doublet; t, triplet; q, quartet; m, multiplet; dd, doublet of doublets; and brs, broad singlet. Elemental analyses (C, H, N) were within ± 0.3% of theoretical values and were determined by Sumika Chemical Analysis Service, Ltd. (Osaka, Japan). Analytical HPLC was conducted using a corona-charged aerosol detector. The column was a L-column 2 octa decyl silyl (ODS) (30 mm × 2.0 mm internal diameter [i.d.]; CERI, Tokyo, Japan) at a temperature of 50 °C and a flow rate of 0.5 mL/min. Mobile phases A and B under neutral conditions were mixtures of 50 mM ammonium acetate (NH_4_OAc), water, and CH_3_CN (1/8/1, *v/v/v*) and 50 mM NH_4_OAc and CH_3_CN (1/9, *v/v*), respectively. The ratio of mobile phase B was increased linearly from 5 to 95% over 3 min, and held at 95% for the next minute. Mobile phases A and B under acidic conditions were a mixture of 0.2% formic acid in 10 mM ammonium formate and 0.2% formic acid in CH_3_CN, respectively. The ratio of mobile phase B was increased linearly from 14 to 86% over 3 min and held at 86% for the next minute.

LC–MS analysis was performed on a Shimadzu (Kyoto, Japan) LC–MS system, operating in electrospray ionization (+ or −) mode. The column used was an L-column 2 ODS (3.0 mm × 50 mm i.d., 3 μm; CERI) with a temperature of 40 °C and a flow rate of 1.2 or 1.5 mL/min. Condition 1: Mobile phases A and B under acidic conditions contained 0.05% TFA in water and 0.05% TFA in CH_3_CN, respectively. The ratio of mobile phase B was increased linearly from 5 to 90% over 0.9 min and held at 90% for the next 1.1 min. Condition 2: Mobile phases A and B under neutral conditions were a mixture of 5 mM NH_4_OAc and CH_3_CN (9/1, *v/v*) and 5 mM NH_4_OAc and CH_3_CN (1/9, *v/v*), respectively. The ratio of mobile phase B was increased linearly from 5 to 90% over 0.9 min and held at 90% for the next 1.1 min. Flash chromatography was performed on silica gel columns (Inject, Hi-Flash or Universal columns, Silica or Amino; Yamazen, Osaka, Japan) or PurifPack (SI or NH, particle size: 60 μm; Fuji Silysia Chemical, Ltd., Aichi, Japan). The reaction progress was determined using TLC on Merck silica gel 60 F_254_ plates. Visualization was performed under UV light (254 nm) or iodine. However, the yields of the purified compounds were not optimized. The reagents and solvents were obtained from commercial sources and used without further purification. All biologically characterized compounds are >95% pure by HPLC.

### Methyl 4-((5-Bromo-3-methyl-1*H*-pyrazol-1-yl)­methyl)­benzoate (**4**)

Cs_2_CO_3_ (24.3 g, 74.5 mmol) was added to a solution of 5-bromo-3-methyl-1*H*-pyrazole (**2**) (10.0 g, 62.1 mmol) in CH_3_CN (200 mL) at rt. After stirring at the same temperature for 10 min, methyl 4-(bromomethyl)­benzoate (**3**) (15.7 g, 68.3 mmol) was added to the reaction mixture. The mixture was then stirred overnight at rt. The mixture was filtered, and the filtrate was concentrated *in vacuo*. The residue was purified using column chromatography (silica gel, eluted with 5–30% ethyl acetate [EtOAc] in hexane) to give the title compound (4.9 g, 15.7 mmol, 25%) as a white solid and the isomer, methyl 4-((3-bromo-5-methyl-1*H*-pyrazol-1-yl)­methyl)­benzoate (13.8 g, 44.6 mmol, 72%), as a white solid.

^1^H NMR (300 MHz, CDCl_3_) of **4**: δ 2.27 (3H, s), 3.90 (3H, s), 5.35 (2H, s), 6.14 (1H, s), 7.19–7.25 (2H, m), 7.92–8.07 (2H, m).

^1^H NMR (300 MHz, CDCl_3_) of the isomer: δ 2.16 (3H, d, *J* = 0.7 Hz), 3.91 (3H, s), 5.29 (2H, s), 6.10 (1H, d, *J* = 0.8 Hz), 7.10–7.20 (2H, m), 7.93–8.06 (2H, m).

### 4-((5-Bromo-3-methyl-1*H*-pyrazol-1-yl)­methyl)­benzoic Acid (**5**)

The 1 M NaOH (31 mL, 31 mmol) was added to a solution of **4** (4.8 g, 15.5 mmol) in a mixture of MeOH and THF (1/1, *v/v*) (100 mL) at rt. The mixture was then stirred at the same temperature for 3 h. The mixture was concentrated *in vacuo*, and 1 M HCl (31 mL) was added to adjust the pH of the solution to 2–3. The precipitate was collected, washed with water, and dried to give the title compound (4.5 g, 15.3 mmol, 98%) as a white solid.

^1^H NMR (300 MHz, DMSO-*d*_6_): δ 2.17 (3H, s), 5.38 (2H, s), 6.31 (1H, s), 7.17–7.25 (2H, m), 7.85–7.97 (2H, m), 12.97 (1H, brs).

### 4-((5-Bromo-3-methyl-1*H*-pyrazol-1-yl)­methyl)-*N*-((tetrahydro-2*H*-pyran-2-yl)­oxy)­benzamide (**6**)

Triethylamine (TEA) (6.4 mL, 45.7 mmol) was added to a solution of **5** (4.5 g, 15.3 mmol), *O*-(tetrahydro-2*H*-pyran-2-yl)­hydroxylamine (2.7 g, 22.9 mmol), 1-hydroxybenzotriazole (HOBt) (3.1 g, 22.9 mmol) and 1-(3-(dimethylamino)­propyl)-3-ethylcarbodiimide hydrochloride (EDC·HCl) (4.4 g, 22.9 mmol) in DMF (50 mL) at rt. The mixture was then stirred overnight at the same temperature. The mixture was then diluted with saturated NaHCO_3_ at rt and extracted with EtOAc. The organic layer was washed with water, saturated NaCl, dried over MgSO_4_, and concentrated *in vacuo*. The residue was purified by column chromatography (silica gel, eluted with 50–100% EtOAc in hexane) to obtain the title compound (5.6 g, 14.1 mmol, 93%) as a white amorphous powder.

^1^H NMR (300 MHz, CDCl_3_) δ 1.53–1.69 (3H, m), 1.78–1.95 (3H, m), 2.26 (3H, s), 3.54–3.71 (1H, m), 3.88–4.04 (1H, m), 5.06 (1H, t, *J* = 2.8 Hz), 5.33 (2H, s), 6.13 (1H, s), 7.15–7.24 (2H, m), 7.64–7.77 (2H, m), 8.92 (1H, s). ESI-MS *m*/*z* 394.0 [M + H]^+^.

### 4-((5-(6-Fluoropyridin-3-yl)-3-methyl-1*H*-pyrazol-1-yl)­methyl)-*N*-hydroxybenzamide (**8**)

PdCl_2_(AmPhos)_2_ (85 mg, 0.1 mmol) was added to a suspension of **6** (500 mg, 1.3 mmol), (6-fluoropyridin-3-yl)­boronic acid (357 mg, 2.5 mmol), and K_2_CO_3_ (526 mg, 3.8 mmol) in a mixture of DME and H_2_O (9/1, *v/v*) (6.3 mL) at rt. The mixture was stirred at 100 °C under N_2_ atmosphere for 24 h. The mixture was then poured into water and extracted using EtOAc. The organic layer was washed with saturated NaCl, dried over MgSO_4_ and concentrated *in vacuo*. The residue was subjected to column chromatography (silica gel, eluted with 50–100% EtOAc in hexane) to obtain a colorless amorphous solid (532 mg) containing 4-((5-(6-fluoropyridin-3-yl)-3-methyl-1*H*-pyrazol-1-yl)­methyl)-*N*-((tetrahydro-2*H*-pyran-2-yl)­oxy)­benzamide (**7**). ESI-MS *m*/*z* 411.2 [M + H]^+^.

The 0.1 M HCl (13.0 mL, 1.3 mmol) was added to a solution of compound **7** (532 mg) in a mixture of MeOH and THF (1/1, *v/v*) (6.5 mL) at 0 °C. The mixture was warmed to rt and stirred for 2 h. The solvent was removed under reduced pressure. The residue was diluted with water and extracted with EtOAc. The extract was washed with saturated NaCl, dried over Na_2_SO_4_, and concentrated *in vacuo*. The residue was crystallized from EtOAc-hexane to give the title compound (217 mg, 665 μmol, 51%) as a white solid.

^1^H NMR (300 MHz, DMSO-*d*_6_) δ 2.22 (3H, s), 5.35 (2H, s), 6.38 (1H, s), 6.97–7.07 (2H, m), 7.24–7.32 (1H, m), 7.60–7.71 (2H, m), 8.00 (1H, td, *J* = 2.5, 8.2 Hz), 8.18–8.32 (1H, m), 9.01 (1H, d, *J* = 1.6 Hz), 11.20 (1H, s). ESI-MS *m*/*z* 327.1 [M + H]^+^.

### *tert*-Butyl 2-((4-(Methoxycarbonyl)­phenyl)­methyl)­hydrazine-1-carboxylate (**9**)

TEA (28.3 g, 279 mmol), followed by methyl 4-(bromomethyl)­benzoate (**3**) (20.0 g, 87.3 mmol), was added to a solution of *tert*-butyl carbazate (23.1 g, 175 mmol) in DMF (140 mL) under N_2_ atmosphere. The mixture was stirred at rt for 1 h and then heated at 75 °C for 5 h. After the completion of the reaction, the mixture was diluted with water and extracted with EtOAc. The extract was dried over Na_2_SO_4_ and concentrated under reduced pressure to produce the title compound (18.2 g, 64.6 mmol, 74%) as a yellow solid.

^1^H NMR (400 MHz, DMSO-*d*_6_) δ 1.37 (9H, s), 3.84 (3H, s), 3.94 (2H, s), 4.92–4.93 (1H, m), 7.47 (2H, d, *J* = 7.8 Hz), 7.89 (2H, d, *J* = 7.9 Hz), 8.26 (1H, brs).

### Methyl 4-((5-Hydroxy-3-methyl-1*H*-pyrazol-1-yl)­methyl)­benzoate (**11**)

The 4 M HCl in 1,4-dioxane (285 mL) was added to an ice-cooled solution of **9** (19.0 g, 67.8 mmol) in 1,4-dioxane (262 mL). The mixture was then warmed to rt and stirred for 2 h. After completion of the reaction monitored by TLC and LC–MS, the solvent was removed under reduced pressure to produce the HCl salt of methyl 4-(hydrazinylmethyl)­benzoate (**10**) (12.2 g, >98%) as a white solid. A mixture of the HCl salt of **10** (3.5 g, 19.4 mmol) and methyl 3-oxobutanoate (2.3 g, 19.4 mmol) in acetic acid (55.0 mL) was heated at 125 °C under N_2_ atmosphere for 4 h. After completion of the reaction monitored by TLC and LC–MS, the solvent was removed under reduced pressure, and the residue was purified using column chromatography (NH_2_ silica gel, eluted with DCM) to produce the title compound (1.4 g, 5.8 mmol, 30%) as a yellow solid.

^1^H NMR (400 MHz, DMSO-*d*_6_) δ 2.00 (3H, s), 3.83 (3H, s), 5.04 (2H, s), 5.20 (1H, s), 7.25 (2H, d, *J* = 6.6 Hz), 7.91 (2H, d, *J* = 7.6 Hz), 10.88 (1H, s).

### Methyl 4-((3-Methyl-5-(((trifluoromethane)­sulfonyl)­oxy)-1*H*-pyrazol-1-yl)­methyl)­benzoate (**12**)

*N*,*N*-Diisopropylethylamine (DIPEA) (2.2 g, 17.1 mmol) and *N*-phenyl-bis­(trifluoromethanesulfonimide) (2.4 g, 6.9 mmol) were added to a solution of **11** (1.4 g, 5.7 mmol) in DCM (56.0 mL). The reaction mixture was refluxed at 40 °C with stirring for 1 h. After the completion of the reaction, which was monitored using TLC and LC–MS, the reaction mixture was diluted with water and extracted with DCM. The extract was then dried over anhydrous Na_2_SO_4_ and concentrated under reduced pressure. The residue was purified by column chromatography (silica gel, eluted with 20% EtOAc in hexane) to obtain the title compound (1.4 g, 3.7 mmol, 64%) as a yellow solid.

^1^H NMR (400 MHz, DMSO-*d*_6_) δ 2.19 (3H, s), 3.84 (3H, s), 5.36 (2H, s), 6.29 (1H, s), 7.29 (2H, d, *J* = 8.0 Hz), 7.94 (2H, d, *J* = 8.0 Hz).

### Methyl 4-((5-(6-Fluoropyridin-2-yl)-3-methyl-1*H*-pyrazol-1-yl)­methyl)­benzoate (**13a**)

A mixture of **12** (1.1 g, 3.0 mmol), 2-(6-fluoropyridine)­cyclic-triolborate lithium salt (1.4 g, 6.1 mmol), bis­(di*tert*-butyl­(4-dimethylaminophenyl)­phosphine)­dichloropalladium­(II) (204 mg, 0.3 mmol), CuCl (150 mg, 1.5 mmol), and K_3_PO_4_ (1.9 g, 9.1 mmol) in a mixture of DME and H_2_O (9/1, *v/v*) (30.0 mL) was heated at 100 °C with stirring under microwave irradiation for 1 h. The mixture was then diluted with water and extracted with EtOAc. The extract was washed with water, saturated NaCl, dried over Na_2_SO_4_, and concentrated *in vacuo*. The residue was purified using column chromatography (NH_2_ silica gel, eluted with 10–35% EtOAc in hexane) to obtain the title compound (585 mg, 1.8 mmol, 59%) as a white solid.

^1^H NMR (300 MHz, CDCl_3_) δ 2.34 (3H, s), 3.86 (3H, s), 5.91 (2H, s), 6.49 (1H, s), 6.74–6.86 (1H, m), 7.18–7.25 (2H, m), 7.38 (1H, ddd, *J* = 7.6, 2.4, 0.7 Hz), 7.70–7.80 (1H, m), 7.86–7.94 (2H, m). ESI-MS *m*/*z* 326.1 [M + H]^+^.

### Methyl 4-((5-(2-Fluoropyridin-4-yl)-3-methyl-1*H*-pyrazol-1-yl)­methyl)­benzoate (**13b**)

K_3_PO_4_ (505 mg, 2.4 mmol) and (2-fluoropyridin-4-yl)­boronic acid (335 mg, 2.4 mmol) were added to a solution of **12** (300 mg, 0.8 mmol) in 1,4-dioxane (7.9 mL) and the mixture was degassed by bubbling with N_2_ for 5 min. Then, 1,1′-bis­(diphenylphosphino)­ferrocene (dppf) (22.0 mg, 39.7 μmol) and PdCl_2_(dppf) (58.0 mg, 79.3 μmol) were added to the reaction vessel. The mixture was heated at 100 °C with stirring under N_2_ atmosphere overnight. The reaction mixture was filtered through a pad of Celite (Fujifilm, Tokyo, Japan), and the filtrate was concentrated under reduced pressure. The residue was diluted with water and extracted with EtOAc. The organic layer was washed with saturated NaCl, dried over MgSO_4_, and concentrated *in vacuo*. The residue was purified using column chromatography (silica gel, eluted with 10–50% EtOAc in hexane) to produce the title compound (232 mg, 713 μmol, 90%) as a colorless oil.

^1^H NMR (400 MHz, DMSO-*d*_6_) δ 2.24 (3H, s), 3.82 (3H, s), 5.53 (2H, s), 6.57 (1H, s), 7.09–7.14 (2H, m), 7.21–7.24 (1H, s), 7.34–7.39 (1H, m), 7.85–7.91 (2H, m), 8.24–8.28 (1H, m). ESI-MS *m*/*z* 326.1 [M + H]^+^.

### Methyl 4-((5-(2-Fluoropyridin-3-yl)-3-methyl-1*H*-pyrazol-1-yl)­methyl)­benzoate (**13c**)

Compound **13c** was prepared from compound **12** as a pale orange oil in 97% yield using a method similar to that described for **13b**.

^1^H NMR (300 MHz, CDCl_3_) δ 2.35 (3H, s), 3.88 (3H, s), 5.29 (2H, s), 6.24 (1H, s), 7.01 (2H, d, *J* = 8.6 Hz), 7.17 (1H, ddd, *J* = 7.2, 5.1, 1.8 Hz), 7.54 (1H, ddd, *J* = 9.3, 7.4, 2.0 Hz), 7.81–7.99 (2H, m), 8.16–8.31 (1H, m). ESI-MS *m*/*z* 326.1 [M + H]^+^.

### 4-((5-(6-Fluoropyridin-2-yl)-3-methyl-1*H*-pyrazol-1-yl)­methyl)­benzoic Acid (**14a**)

TEA (5.1 mL, 36.9 mmol) was added to a solution of **13a** (600 mg, 1.8 mmol) in a mixture of MeOH and H_2_O (1/1, *v/v*) (20.0 mL) at rt. The mixture was heated at 70 °C with stirring under N_2_ atmosphere for 48 h. The reaction mixture was then concentrated under reduced pressure and diluted with 1 M HCl. The resulting precipitate was collected using filtration, washed with water, and dried under reduced pressure to obtain the title compound (540 mg, 1.7 mmol, 94%) as a white solid. The product was used in the next step without further purification.

^1^H NMR (300 MHz, DMSO-*d*_6_) δ 2.23 (3H, s), 5.84 (2H, s), 6.79 (1H, s), 7.12 (1H, dd, *J* = 8.0, 2.5 Hz), 7.18 (2H, d, *J* = 8.5 Hz), 7.72 (1H, dd, *J* = 7.4, 2.3 Hz), 7.80–7.89 (2H, m), 7.98–8.11 (1H, m), 12.87 (1H, s). ESI-MS *m*/*z* 312.1 [M + H]^+^.

### 4-((5-(2-Fluoropyridin-4-yl)-3-methyl-1*H*-pyrazol-1-yl)­methyl)­benzoic Acid (**14b**)

Potassium trimethylsilanolate (137 mg, 1.1 mmol) was added to an ice-cooled solution of **13b** (232 mg, 0.7 mmol) in THF (3.6 mL). The mixture was warmed to rt and stirred under N_2_ atmosphere overnight. The solvent was then removed by evaporation under reduced pressure. The residue was dissolved in a minimum volume of water, cooled to 0 °C, and acidified with 1 M HCl. The aqueous mixture was extracted using EtOAc. The extract was washed with saturated NaCl, dried over MgSO_4_, and concentrated *in vacuo* to give the title compound (203 mg, 652 μmol, 91%) as a white solid. The product was used in the next step without further purification.

^1^H NMR (400 MHz, DMSO-*d*_6_) δ 2.24 (3H, s), 5.52 (2H, s), 6.56 (1H, s), 7.06–7.12 (2H, m), 7.22–7.24 (1H, m), 7.36–7.39 (1H, m), 7.82–7.89 (2H, m), 8.26–8.28 (1H, m), 12.92 (1H, brs). ESI-MS *m*/*z* 312.1 [M + H]^+^.

### 4-((5-(2-Fluoropyridin-3-yl)-3-methyl-1*H*-pyrazol-1-yl)­methyl)­benzoic Acid (**14c**)

Compound **14c** was prepared from compound **13c** as a white solid in 84% yield by a method similar to that described for **14b**.

^1^H NMR (300 MHz, DMSO-*d*_6_) δ 2.23 (3H, s), 5.29 (2H, s), 6.35 (1H, s), 6.97–7.09 (2H,m), 7.43 (1H, ddd, *J* = 7.3, 5.0, 1.9 Hz), 7.82 (2H, d, *J* = 8.4 Hz), 7.93 (1H, ddd, *J* = 9.7, 7.6, 2.0 Hz), 8.26–8.34 (1H, m), 12.92 (1H, brs). ESI-MS *m*/*z* 312.1 [M + H]^+^.

### 4-((5-(6-Fluoropyridin-2-yl)-3-methyl-1*H*-pyrazol-1-yl)­methyl)-*N*-hydroxybenzamide (**16a**)

TEA (675 μL, 4.8 mmol) was added a solution of **14a** (540 mg, 1.6 mmol), *O*-(tetrahydro-2*H*-pyran-2-yl)­hydroxylamine (283 mg, 2.4 mmol), HOBt (327 mg, 2.4 mmol), and EDC·HCl (464 mg, 2.4 mmol) in DMF (20 mL) at rt. The mixture was then stirred overnight at the same temperature. The mixture was then diluted with saturated NaHCO_3_ and extracted with EtOAc. The extract was washed with water, saturated NaCl, dried over MgSO_4_, and concentrated *in vacuo*. The residue was purified by column chromatography (silica gel, eluted with 50–100% EtOAc in hexane) to give 4-((5-(6-fluoropyridin-2-yl)-3-methyl-1*H*-pyrazol-1-yl)­methyl)-*N*-((tetrahydro-2*H*-pyran-2-yl)­oxy)­benzamide (**15a**) (570 mg, 1.4 mmol, 86%) as a white amorphous powder. ESI MS *m*/*z* 411.3 [M + H]^+^.

The 0.1 M HCl (13.6 mL, 1.4 mmol) was added to a solution of **15a** (560 mg, 1.4 mmol) in a mixture of MeOH and THF (1/1, v/v) (30.0 mL) at rt. The mixture was then stirred overnight at the same temperature. The reaction mixture was then concentrated *in vacuo*. The residue was crystallized from MeOH to produce the title compound (280 mg, 858 μmol, 63%) as a white solid.

^1^H NMR (300 MHz, DMSO-*d*_6_) δ 2.22 (3H, s), 5.81 (2H, s), 6.78 (1H, s), 7.07–7.20 (3H, m), 7.60–7.66 (2H, m), 7.72 (1H, dd, *J* = 7.6, 2.6 Hz), 7.95–8.16 (1H, m), 8.98 (1H, brs), 11.11 (1H, s). ESI-MS *m*/*z* 327.1 [M + H]^+^. Anal. Calcd for C_17_H_15_FN_4_O_2_: C, 62.57; H, 4.63; N, 17.17. Found: C, 62.60; H, 4.60; N, 17.15.

### 4-((5-(2-Fluoropyridin-4-yl)-3-methyl-1*H*-pyrazol-1-yl)­methyl)-*N*-hydroxybenzamide (**16b**)

Compound **16b** was prepared from compound **14b** as a white solid in 43% yield (two steps) by a method similar to that described for **16a**.

^1^H NMR (300 MHz, DMSO-*d*_6_) δ 2.23 (3H, s), 5.49 (2H, s), 6.56 (1H, s), 7.05 (2H, d, *J* = 8.3 Hz), 7.25 (1H, s), 7.40 (1H, dt, *J* = 5.3, 1.7 Hz), 7.66 (2 H, d, *J* = 8.5 Hz), 8.28 (1 H, d, *J* = 5.1 Hz), 9.03 (1H, brs), 11.17 (1 H, brs). ESI-MS *m*/*z* 327.1 [M + H]^+^.

### 4-((5-(2-Fluoropyridin-3-yl)-3-methyl-1*H*-pyrazol-1-yl)­methyl)-*N*-hydroxybenzamide (**16c**)

Compound **16c** was prepared from compound **14c** as a white solid in 18% yield (two steps) by a method similar to that described for **16a**.

^1^H NMR (300 MHz, DMSO-*d*_6_) δ 2.23 (3H, s), 5.24 (2H, s), 6.34 (1H, s), 6.99 (2H, d, *J* = 8.3 Hz), 7.44 (1H, ddd, *J* = 7.3, 5.0, 1.9 Hz), 7.62 (2H, d, *J* = 8.4 Hz), 7.94 (1H, ddd, *J* = 9.7, 7.6, 2.0 Hz), 8.22–8.38 (1H, m), 9.00 (1H, s), 11.15 (1H, brs). ESI-MS *m*/*z* 327.1 [M + H]^+^.

### 4-((3-Methyl-5-(6-nitropyridin-2-yl)-1*H*-pyrazol-1-yl)­methyl)-*N*-((tetrahydro-2*H*-pyran-2-yl)­oxy)­benzamide (**19**)

PdCl_2_(PCy_3_)_2_ (114 mg, 0.2 mmol) was added to a solution of **4** (500 mg, 1.6 mmol), bis­(pinacolato)­diboron (821 mg, 3.2 mmol) and KOAc (476 mg, 4.9 mmol) in cyclopentyl methyl ether (16.9 mL) at rt. The mixture was heated at 100 °C with stirring for 4 h. Then, 1 M K_2_CO_3_ (3.2 mL, 3.2 mmol), 2-chloro-6-nitropyridine (513 mg, 3.2 mmol), and PdCl_2_(PCy_3_)_2_ (114 mg, 0.2 mmol) were added. The mixture was heated at 100 °C for 2 h. The mixture was then poured into water and extracted using EtOAc. The extract was washed with saturated NaCl, dried over MgSO_4_, and concentrated *in vacuo*. The residue was purified using column chromatography (silica gel, eluted with 25–50% EtOAc in hexane) to give methyl 4-((3-methyl-5-(6-nitropyridin-2-yl)-1*H*-pyrazol-1-yl)­methyl)­benzoate (**17**) (487 mg, 1.4 mmol, 85%) as a white solid. ESI-MS *m*/*z* 353.1 [M + H]^+^.

The 1 M NaOH (1.4 mL, 1.4 mmol) was added to a solution of **17** (487 mg, 1.4 mmol) in a mixture of THF and H_2_O (1/1, *v/v*) (14.7 mL). The mixture was heated at 80 °C with stirring overnight. The organic solvent was removed under reduced pressure. The aqueous solution was then acidified with 1 M HCl and extracted with EtOAc. The extract was washed with saturated NaCl, dried over MgSO_4_, and concentrated *in vacuo* to produce 4-((3-methyl-5-(6-nitropyridin-2-yl)-1*H*-pyrazol-1-yl)­methyl)­benzoic acid (**18**) (418 mg, 1.2 mmol, 89%) as a light brown solid. ESI-MS *m*/*z* 339.1 [M + H]^+^.

EDC·HCl (355 mg, 1.9 mmol), HOBt (250 mg, 1.9 mmol), and DIPEA (1.1 mL, 6.2 mmol) were added to an ice-cooled solution of **18** (418 mg, 1.2 mmol) in DMF (6.2 mL). After the mixture was stirred at 0 °C for 5 min, *O*-(tetrahydro-2*H*-pyran-2-yl)­hydroxylamine (217 mg, 1.9 mmol) was added. Subsequently, the mixture was warmed to rt and stirred overnight. It was then diluted with saturated NH_4_Cl and extracted with EtOAc. The extract was washed with saturated NaCl, dried over MgSO_4_, and subsequently concentrated *in vacuo*. The residue was purified through column chromatography (silica gel, eluted with 50–100% EtOAc in hexane) to yield the title compound (256 mg, 585 μmol, 47%) in the form of a white solid. ^1^H NMR (300 MHz, DMSO-*d*_6_) δ 1.41–1.84 (6H, m), 2.25 (3H, s), 3.43–3.55 (1H, m), 3.94–4.09 (1H, m), 4.90–4.99 (1H, m), 5.99 (2H, s), 6.96 (1H, s), 7.24 (2H, d, *J* = 8.4 Hz), 7.59–7.66 (2H, m), 8.19–8.34 (3H, m), 11.53 (1H, s). ESI-MS *m*/*z* 438.2 [M + H]^+^.

### 4-((5-(6-[^18^F]­Fluoropyridin-2-yl)-3-methyl-*1H*-pyrazol-1-yl)­methyl)-*N*-hydroxybenzamide ([^18^F]**16a**)

A total of 9.3 GBq of [^18^F]­fluoride was synthesized using a cyclotron through the ^18^O­(p, n)^18^F reaction on 98 atom % H_2_^18^O (Rotem Industries, Arava, Israel). The cyclotron-generated [^18^F]­HF was isolated from H_2_^18^O using a Sep-Pak Accell Plus QMA Plus Light cartridge (Waters, Milford, MA, USA), which was pretreated by passing 10 mL of water through it, followed by purging with nitrogen gas to remove excess moisture before use. The resultant [^18^F]­HF was eluted from the cartridge with a mixture of aqueous K_2_CO_3_ (2.8 mg/0.2 mL) and a solution of 4,7,13,16,21,24-hexaoxa-1,10-diazabicyclo­[8,8,8]-hexacosane (7.5 mg) in CH_3_CN (0.2 mL) and transferred into a reaction vessel within the hot cell. The [^18^F]­KF solution was dried at 120 °C for 30 min to remove water and CH_3_CN. To the vessel containing [^18^F]­KF, a solution of the nitro precursor **19** (1.0 mg) in anhydrous DMSO (300 μL) was added and heated at 150 °C for 10 min. After [^18^F]­fluorination of **19** with [^18^F]­KF, 1 M HCl (300 μL) was added into the reaction mixture and heated at 90 °C for 4 min. The resultant reaction mixture was then diluted with a solution of CH_3_CN, H_2_O, and TFA (25/75/0.1, *v/v/v*, 0.5 mL) and injected into an HPLC column for further analysis. HPLC purification was performed on a CAPCELL PAK C18 column (10 mm i.d. × 250 mm; Osaka Soda, Osaka, Japan) using a mobile phase of CH_3_CN and 0.1% TFA in water (25/75, *v/v*) at a flow rate of 4.0 mL/min. The radioactive fraction corresponding to [^18^F]**16a** (^*t*^R = 26.5 min) was collected in a sterile flask containing polysorbate 80 (100 μL) and 25% ascorbic acid (100 μL), then evaporated to dryness under vacuum, redissolved in 3 mL of sterile saline, and subsequently passed through a 0.22 μm Millipore filter to obtain the final product. The analytical HPLC was performed on a CAPCELL PAK C18 column (4.6 mm i.d. × 150 mm; Osaka Soda) using a mobile phase of CH_3_CN and 0.1% TFA in water (30/70, *v/v*) at a flow rate of 1.0 mL/min. The identity of [^18^F]**16a** was established through coinjection with **16a** (^*t*^R = 9.7 min). The total synthesis time was 86 min from EOB, resulting in a radiochemical yield (decay-corrected) of 32% based on [^18^F]­fluoride, radiochemical purity of >99%, and molar activity at the end of synthesis of 426 GBq/μmol.

### HDAC Enzyme Assay and Selectivity

The assay was performed by Axcelead Drug Discovery Partners, Inc. (Kanagawa, Japan). Recombinant human HDAC6, HDAC7, and HDAC1 were obtained from SignalChem Pharmaceutical Inc. (Richmond, BC, Canada), recombinant HDAC8 was obtained from Reaction Biology Corporation (Marvern, PA, USA), and recombinant human HDAC4 and mouse HDAC6 were prepared by Axcelead Drug Discovery Partners Inc. A test compound (1 μL) diluted with DMSO was added to 75 μL of assay buffer (24 mM Tris-HCl [pH 7.5], 135 mM NaCl, 0.35 mM KCl, 1 mM MgCl_2_, 0.01% Tween 20, 0.6 mM glutathione) in 384 well plates. Subsequently, 2 μL of diluted compounds was added to 384 well plates, and 2 μL of recombinant HDAC enzymes was added and then centrifuged (Himac CT9RX; HITACHI, Tokyo, Japan) at 240 *g* for 10 s. The final concentrations of each HDAC enzyme were as follows: human HDAC1, 170 pM; human HDAC6, 2.8 pM; human HDAC4, 68 pM; human HDAC7, 13 pM; human HDAC8, 5 nM; mouse HDAC6, 323 pM. After incubation for 60 min at rt, 4 μL of HDAC-Glo reagent (HDAC-Glo I/II [Promega] for HDAC6, HDAC1, and HDAC8 and HDAC-Glo class IIa [Promega] for HDAC4 and HDAC7) were added to start the reaction. After incubation for 20 min at rt, the luminescence was measured using an Envision plate reader (PerkinElmer, Waltham, MA, USA). To determine inhibitory activity, the vehicle signal was set at 0%, and the signal in the absence of HDAC enzyme was set at 100% inhibition. The inhibitory activity was expressed as the value of IC_50_. The IC_50_ values and 95% confidence intervals were calculated using XLfit (IDBS Software Solutions, Surrey, U.K.).

### Measurement of Log *D*_7.4_

Log *D*_7.4_, which is the partition coefficient between 1-octanol and aqueous buffer (pH 7.4) of the compounds, was measured using a chromatographic procedure whose condition was developed based on a published method.^19,20^

### Measurement of MDR1 Membrane Permeability

Human MDR1-expressing LLC-PK1 cells were cultured, and the transcellular transport study was performed with minor modifications to the previously reported method.^21^ Specifically, the cells were grown in Transwell 96-well permeable support (pore size, 0.4 μm; surface area, 0.143 cm^2^) with polycarbonate membrane (Corning Life Sciences, Lowell, MA, USA). The cells were preincubated with M199 at 37 °C. Subsequently, transcellular transport was initiated by the addition of M199 either to apical compartments (75 μL) or to basolateral compartments (250 μL) containing 1 μM test compounds. The assay was terminated by removing each assay plate after 1 h. Aliquots (25 μL) from the opposite compartments were mixed with CH_3_CN and then centrifuged. The concentrations of the compounds in the supernatant were measured through LC–MS/MS. The apparent permeability (*P*_app_) of the test compounds in the receiver wells was determined, and the efflux ratio (ER) for the MDR1 membrane permeability test was calculated as follows:

$$\mathrm{ER}=P_{\mathrm{app},\mathrm{BtoA}}/P_{\mathrm{app},\mathrm{AtoB}}$$

where *P*_app,AtoB_ and *P*_app,BtoA_ represent the apparent permeabilities in the apical-to-basal and basal-to-apical directions, respectively.

### AS-MS Direct Binding Assay

The binding assay using AS-MS was performed at rt in a final volume of 10 μL using the recombinant HDAC6 (HDAC6, Active, H88-30G-10, Lot:B2010-9; SignalChem Biotech Inc., Richmond, BC, Canada). The protein (3 nM) was incubated with test compound at eight serially diluted concentrations for 1 h in assay buffer (50 mM 4-(2-hydroxyethyl)-1-piperazineethanesulfonic acid [HEPES] [pH 7.3], 10 mM MgCl_2_, 1 mM ethylene glycol tetraacetic acid [EGTA], and 0.005% Tween 20). For estimating nonspecific binding, excess amounts of competitor compounds (10 μM Tubastatin A) were added to the mixture of HDAC6 and test compounds. The reaction was terminated by separating the bound and free compounds at 1 h using a 384-well filtration membrane (5085; Pall Corp, NY, USA) packed with a gel filtration resin. Subsequently, 5 μL of flow-through was mixed with 50 μL of water, CH_3_CN, and MeOH (6/1/1, v/v/v) containing 1% formic acid to denature the protein–compound complexes. The liberated compounds were quantified through LC-MS using an Agilent 6495 RapidFire/MS/MS system equipped with an ESI interface (Agilent, CA, USA). The compounds were separated on a reverse-phase column (C18; Agilent, CA, USA). The mobile phase consisted of solvents A (10 mM ammonium formate containing 0.2% formic acid) and solvent B (CH_3_CN containing 0.2% formic acid). The mass transitions (Q1/Q3) used for **16a** and bavarostat were *m*/*z* 327.13/118 and 347.22/149.2, respectively.

### AS-MS Dissociation Assay

The binding assay using AS-MS was performed at rt in a final volume of 60 μL using the recombinant HDAC6 (HDAC6, Active, H88-30G-10, Lot:B2010-9; SignalChem Biotech Inc.). The protein (3 nM) was incubated with test compounds at a concentration of its apparent equilibrium dissociation constant (*K*_d_) for HDAC6 for 1 h in assay buffer (50 mM HEPES [pH 7.3], 10 mM MgCl_2_, 1 mM EGTA, and 0.005% Tween 20). After incubation, excess amounts of competitor compounds (10 μM Tubastatin A) were added to initiate dissociation of the test compounds. The reaction was terminated by separating the bound and free compounds at each time point using a 384-well filtration membrane (5085; Pall Corp.) packed with a gel filtration resin. Subsequently, 5 μL of flow-through was mixed with 30 μL of water, acetonitrile, and MeOH (6/1/1, v/v/v) containing 1% formic acid to denature the protein–compound complexes. The liberated compounds were quantified through LC-MS using an Agilent6495 LC–MS/MS system equipped with an ESI interface (Agilent, CA, USA). The compounds were separated on a reversed-phase column (C18; Agilent Technologies). The mobile phase consisted of solvents A (10 mM ammonium formate containing 0.2% formic acid) and B (CH_3_CN containing 0.2% formic acid). The mass transitions (Q1/Q3) used for **16a** and bavarostat were *m*/*z* 327.13/118 and 347.22/149.2, respectively.

### AS-MS Data Analysis

All data were analyzed using Prism 5 (version 6.07; GraphPad Software, Boston, MA, USA). The binding rate constants and *K*_d_ were calculated using GraphPad Prism software using the nonlinear iterative curve-fitting computer program, employing the following equation:

$$Y=B_{\max}\times X/\left(K_{d}+X\right)$$

To simplify the analysis, we assumed that the amounts of the probe and competitor bound to the receptor were considerably lower than their free concentrations. The concentrations of the free probe and competitor did not change significantly throughout the experiments. The dissociation binding data were analyzed by fitting to a one-phase exponential decay model:

$$Y=\mathrm{Span}\times \exp\left(-k_{\mathrm{off}}\times X\right)+\mathrm{Plateau}$$

The dissociation half-life (*T*_1/2_) was determined using the following equation:

$$T_{1/2}=\ln\left(2\right)/k_{\mathrm{off}}$$

*k*_on_ was determined using the following equation:

$$k_{\mathrm{on}}=k_{\mathrm{off}}/K_{d}$$

### Unbound Fraction Rate in Mouse Brain

The brain tissue binding of each compound was determined via the equilibrium dialysis method using HTDialysis Teflon dialysis chambers and cellulose membranes (MWCO 6–8 kDa). Brain homogenate was mixed with the compound solution for a final concentration of 1 μM. Dialysis was conducted against PBS at 37 °C for 16–20 h. The concentrations of the compounds in both the biological samples and PBS were determined through LC–MS/MS. The unbound fraction in the biological sample (*F*_u,b_) was calculated as the ratio of the peak area of the compounds from the PBS side to that from the brain homogenate side of the dialysis apparatus.

### Ethics Statement

All studies were reviewed and approved by the Institutional Animal Care and Use Committee (IACUC) of Takeda Pharmaceutical Company Limited (Ethics Approval Number: AU00021083, AU00010849, AU-00011134, and AU-00010058) or the Animal Ethics Committee of the National Institutes for Quantum Science and Technology (Ethics Approval Number: 07-1051-19), accredited by Association for Assessment and Accreditation of Laboratory Animal Care International (AAALAC).

### Animals

Male C57BL/6J mice (7 weeks old) were purchased from CLEA Japan, Inc. (Tokyo, Japan). Male HDAC6 KO mice (36 weeks old) were supplied by Axcelead Drug Discovery Partners Inc., as described in our previous report.^13^ Sprague–Dawley (SD) rats were obtained from Japan SLC (Shizuoka, Japan). They were housed in groups, kept on a 12-h light/dark cycle, and provided ad libitum access to food and water. After acclimation for at least 1 week, the animals were used for the experiments. Male cynomolgus (Macaca fascicularis) and rhesus monkeys (Macaca mulatta) were purchased from Shin Nippon Biomedical Laboratories, Ltd. (Kagoshima, Japan). They were housed individually under a 12-h light/dark cycle and fed once daily with ad libitum access to water. All nonhuman primates were housed and managed in strict adherence to the guidelines for ethical animal handling under the supervision of veterinary professionals. They also received environmental enrichment aimed at promoting their well-being, with vigilant monitoring for any indications of illness, such as changes in attitude, appetite, or behavior. For sampling, the animals were anesthetized and euthanized by exsanguination.

### Reagents in IHC and Western Blotting study

The following antibodies were used in this study: anti-HDAC6 (D21B10 and D2E5; Cell Signaling Technology, Danvers, MA, USA). Recombinant GST-HDAC6 was purchased from SignalChem Biotech, Inc. and used as a positive control for Western blotting.

### IHC Procedure

A male cynomolgus monkey (8 years old, 7.2 kg) was sacrificed under anesthesia and perfused with saline through the carotid artery. The dissected brain was cut into small blocks and meticulously preserved within a solution of 4% paraformaldehyde-PBS for 24 h. IHC staining was performed on 4-μm paraffin sections of monkey brain using an automatic immunostainer VENTANA DISCOVERY XT (Ventana Medical Systems, Inc., Oro Valley, AZ, USA) and anti-HDAC6 antibody, together with DAB Map Detection Kit. The stained images were captured using NanoZoomer 2.0-HT Slide Scanner (Hamamatsu Photonics, Shizuoka, Japan).

### Western Blotting

Brain samples were separated via SDS-PAGE using a NuPAGE 4–12% Bis-Tris Gel (ThermoFisher; Waltham, MA, USA), then electrophoretically transferred to iBlot Gel Transfer Stacks Nitrocellulose (ThermoFisher), and blocked for 30 min in Blockace (DS Pharma Biomedical, Osaka, Japan). After blocking, the membranes were probed with primary antibodies, followed by labeling with horseradish peroxidase-coupled secondary antibodies (Abcam, Cambridge, U.K.), and then visualized using a chemiluminescence reagent (SuperSignal West Femto Maximum Sensitivity Substrate, Thermo Fisher) using ImageQuant LAS 4000 (GE Healthcare Bio-Sciences, Marlborough, MA, USA). Quantitative densitometric analysis was performed using an ImageQuant TL (GE Healthcare Biosciences).

### Mouse Brain Uptake Study

Male HDAC6 KO mice and their littermates (36 weeks old, 35–38 g, *n* = 4) were used in the study. Compound **16a** was reconstituted in 25% DMA in saline and administered intravenously at 1 mg/kg. The mice were sacrificed 2 h after administration. The compound concentration was measured using a previously reported method with modifications.^22^ Briefly, the hippocampal tissues from treated mice were isolated and homogenized in saline under ice-cold conditions and stored at −80 °C until LC–MS/MS analysis. Plasma was obtained with ethylenediaminetetraacetic acid (EDTA) as an anticoagulant and centrifugation at 10,000 g for 15 min and stored at −80 °C. Before the analysis, the samples were thawed gently on ice and mixed with CH_3_CN. The CH_3_CN mixture was vortexed and centrifuged to collect the supernatant. The supernatant was diluted in mobile phase A (10 mM ammonium formate and formic acid at 100/0.2, v/v) and injected into an LC–MS/MS system. The compound was analyzed in multiple reaction monitoring mode using an API4000 or API5000 instrument (Applied Biosystems, Foster City, CA, USA) equipped with a Prominence UHPLC (Shimadzu) using a Shimadzu Shim-pack XR-ODS (2.2 μm, 2.0 mm i.d. × 30 mm) column. Mobile phase B comprised a mixture of CH_3_CN and formic acid at 100/0.2 (v/v). The Analyst software (version 1.6.2; AB SCIEX, Tokyo, Japan) was used for data acquisition and processing.

### *In Vitro* Autoradiography Using Rat Brain Sections

Brain sections were prepared from three SD rats (8–10 weeks old). The rats were killed by decapitation under anesthesia and brains were quickly removed and frozen on powdered dry ice. Sagittal brain sections (20 μm) were cutoff using a cryostat (NX-70, Thermo Fisher Scientific, MA, USA) and mounted on glass slides (Matsunami, Tokyo, Japan). After preincubation with a 50 mM Tris-HCl buffer solution (pH 7.4) containing 2 mM MgCl_2_ and 1.2 mM CaCl_2_ for 20 min at rt, the brain sections were incubated in buffer containing [^18^F]**16**a (4.7 MBq/L, 0.01 nM) for 60 min at rt. For blocking study, unlabeled compound (1 μM) dissolved in DMSO (final conc.: 0.1%, v/v) was coincubated with [^18^F]**16a** solution. Radioactive signals exposed on an imaging plate were analyzed using a Bio Imaging Analyzer System (BAS5000, FujiFilm). The radioactivities in the cerebral cortex, striatum, hippocampus, thalamus, cerebellum, and pons on each section were measured using the software (Multi Gauge, FujiFilm) and expressed as photostimulated luminescence (PSL)/mm^2^.

### PET Assessments Using a Rhesus Monkey

Two PET scans were acquired at 6 weeks interval using the same male monkey (male 8 years old, 6–7 kg). Each monkey underwent one baseline scan with [^18^F]**16a** and a blocking scan at 5 min after the injection of the HDAC6 inhibitor ACY-775 (2 mg/kg). A monkey was anesthetized with intramuscular administration of ketamine (5–10 mg/kg) and xylazine (0.2–0.5 mg/kg) and then maintained in an anesthetized state with 1–2% isoflurane in oxygen. The heart rate, body temperature, end-tidal CO_2_, respiration rate, oxygen saturation, and respiration rate were continuously monitored using Life Scope VS (Nihonkohden, Tokyo). An arterial line was created in the saphenous artery for blood sampling. All PET scans were performed using a Focus 220 PET camera (Siemens Medical Solutions, Knoxville, TN, USA) designed for laboratory animals, which yields a 258 mm diameter × 76 mm axial field of view (FOV) and spatial resolution of 1.3 mm full width at half-maximum at the center of FOV.^23^ A bolus of [^18^F]**16a** (178–207 MBq, 0.5–0.8 nmol) was intravenously injected into a monkey, and dynamic emission scan in 3-D acquisition mode was performed for 120 min (frames × time: 6 × 10 s, 6 × 30 s, 12 × 1 min, 18 × 3 min, and 10 × 5 min). All the list-mode data were sorted into 3D sinograms, which were then Fourier-rebinned into 2-D sinograms. Subsequently, images were reconstructed with filtered back-projection using a Hanning filter with a cutoff at the Nyquist frequency (0.5 mm^–1^). The PET images were coregistered to the NIMH Macaque template.^24^ The ROIs were placed on the dorsolateral frontal cortex, white matter, putamen, caudate nucleus, hippocampus, amygdala, cerebellum, brain stem (including midbrain, pons, and medulla), lateral temporal cortex, and corpus callosum using PMOD image analysis software (version 3.407; PMOD Technologies, Zurich, Switzerland) with reference to the NIMH Macaque template. The TACs of [^18^F]**16a** in these regions were calculated and represented as SUV. For the blocking study, ACY-775 (2 mg/kg dissolved in a solution containing DMSO, Tween 80, and saline, 1/1/8, v/v/v) was injected 5 min before [^18^F]**16a** injection. To obtain an arterial input function, plasma activity and parent fraction over time were measured as previously described.^25^ The serial 24 blood samples (1–2.5 mL) were collected from the saphenous artery throughout the emission scan after [^18^F]**16a** injection. An aliquot of plasma was separated using a refrigerated centrifuge (15,000*g*, 3 min, 4 °C). Radioactivity in whole blood and plasma was measured using a 2480 WIZARD autogamma counter (PerkinElmer, Waltham, MA, USA). In addition to measuring radioactivity concentration, plasma samples collected at 1, 5, 15, 30, 60, 90, and 120 min were used to quantify the fraction of unchanged [^18^F]**16a**. All samples were centrifuged and treated, as previously reported.^25^ An aliquot of the supernatant (100–300 μL) prepared from the plasma was analyzed according to the following HPLC condition: column, CAPCELL PAK C18 (5 μm, 4.6 mm i.d. × 250 mm, Osaka soda); mobile phase, CH_3_CN/H_2_O containing 0.1% TFA (30/70, v/v); flow rate, 1.0 mL/min; and detection wavelength, 254 nm. The ratio of unchanged [^18^F]**16a** to the total radioactivity (corrected for decay) was calculated according to the peak areas in the HPLC chromatogram. The metabolite-corrected arterial plasma input function was then determined using plasma radioactivity concentrations and unmetabolized fractions. The *V*_T_ was estimated through Logan GA (t* = 30 min) using the TACs for these ROIs and the metabolite-corrected input function.^18^ Target occupancy of ACY-775 pretreatment was estimated using Lassen plot as follows:

$$V_{T}^{\mathrm{baseline}}-V_{T}^{\mathrm{ACY}775}=\mathrm{Occ}\left(V_{T}^{\mathrm{baseline}}-V_{\mathrm{ND}}\right)$$

where *V*_T_^baseline^ and *V*_T_^ACY775^ are regional *V*_T_ values at baseline and after the ACY-775 treatment, respectively; *V*_ND_ is nondisplaceable distribution volume; and Occ is occupancy.^26^ The *V*_T_-based parametric images were also estimated through voxel-based Logan GA. Kinetic analysis and reconstruction of parametric PET images were performed using PMOD software.

## Supplementary Material





## Notes

Crystal data for **4**. HPLC chromatograms of [^18^F]**16a**; metabolite-collected plasma input function in NHP PET imaging; and radio-HPLC chromatograms in metabolite analysis (PDF)
Molecular formula strings (CSV)

## Abbreviations Used

AS-MS: affinity selection-mass spectrometry
CNS: central nervous system
DIPEA: *N,N*-diisopropylethylamine
dppf: 1,1′-bis­(diphenylphosphino)­ferrocene
EDC·HCl: 1-ethyl-3-(3-(dimethylamino)­propyl)­carbodiimide hydrochloride
EGTA: ethylene glycol tetraacetic acid
EOB: end of bombardment
EtOAc: ethyl acetate
HDAC6: histone deacetylase 6
HEPES: 4-(2-hydroxyethyl)-1-piperazineethanesulfonic acid
HOBt: 1-hydroxybenzotriazole
i.d.: internal diameter
*K*_d_: dissociation constant
KO: knockout
LC–MS/MS: liquid chromatography-tandem mass spectrometry
MDR1: multidrug resistance protein 1
NH_4_OAc: ammonium acetate
NHP: nonhuman primate
ODS: octa decyl silyl
PET: positron emission tomography
ROI: region of interest
SE: standard error
SUV: standardized uptake value
TAC: time-activity curve
TEA: triethylamine
*V*_T_: distribution volume
