# Supplementary material for: Discovery of Pyrazole-Based Positron Emission Tomography Agent that Maps Histone Deacetylase 6 (HDAC6) in the Nonhuman Primate Brain
Source: J Med Chem. 2025 Oct 21;68(21):23375–88. doi: 10.1021/acs.jmedchem.5c02216 (PMC12621258; doi:10.1021/acs.jmedchem.5c02216)
Supplement: Supplementary file 1 [file jm5c02216_si_001.pdf]

Supporting Information

Discovery of Pyrazole-Based Positron Emission Tomography Agent that Maps Histone Deacetylase 6 (HDAC6) in the Nonhuman Primate Brain

Tomoteru Yamasaki,[a,] Norio Ohyabu,[b]* Takeshi Wakabayashi,[b] Ignacio Ibáñez,[b] Kouichi Iwanaga,[b] Satoshi Yamamoto,[b] Masahiko Hattori,[b] Taku Sugita,[b] Michiko Terada,[b] Tomohiro Onishi,[b] Sho Sato,[b] Yohei Kosugi,[b] Akihiro Takano,[c] Paul McQuade,[d] Takamitsu Maru,[e] Naomi Inui,[e] Masayuki Fujinaga,[a] Wakana Mori,[a] Yuji Nagai,[a] Chie Seki,[a] Shoko Uchida,[a] Takafumi Minamimoto,[a] Makoto Higuchi,[a] Makoto Fushimi,[b] and Ming-Rong Zhang[a]*

[a]*National Institute for Quantum Science and Technology, Inage-ku, Chiba 263-8555, Japan*

[b]*Takeda Pharmaceutical Company Limited, Fujisawa, Kanagawa 251-8555, Japan*

[c]*Takeda Pharmaceutical Company Limited, Chuo-ku, Osaka 540-8645, Japan*

[d]*Takeda Development Center Americas, Inc., Cambridge, Massachusetts 02142, United States*

[e]*Axcelead Drug Discovery Partners, Inc., Fujisawa, Kanagawa 251-0012, Japan*

*To whom correspondence should be addressed. E-mail: norio.ooyabu@takeda.com and zhang.ming-rong@qst.go.jp

Contents

1. **X-ray structure analysis** S3
2. **HPLC chromatograms in radiosynthesis of [^{18}F]16a** S4
3. **Metabolite-corrected plasma input function in NHP PET imaging** S5
4. **Representative radio-HPLC chromatograms in the plasma of baseline subject** S6
5. **References** S7

1. X-ray structure analysis

Crystal data for **4**: $C_{13}H_{13}BrN_2O_2$, *MW* = 309.16; crystal size, 0.21 × 0.02 × 0.01 mm; colourless, needle; monoclinic, space group $P2_1/c$, a = 19.6419(8) Å, b = 4.3225(2) Å, c = 31.2378(15) Å, $\alpha = \gamma$ = 90°, β = 97.917(4)°, V = 2626.9(2) Å3, Z = 8, Dx = 1.563 g/cm3, T = 273 K, μ = 4.246 mm-1, λ = 1.54187 Å, R_1 = 0.0516, wR_2 = 0.1553, S = 1.103.

All measurements were made on a Rigaku XtaLAB P200 diffractometer using multi-layer mirror monochromated Cu-Kα radiation. The structure was solved by direct methods with SIR2008[1] and was refined using full-matrix least-squares on F^2 with SHELXL-2014/7.[2] All non-H atoms were refined with anisotropic displacement parameters.

CCDC 2457646 for compound **4** contains the supplementary crystallographic data for this paper. These data can be obtained free of charge from The Cambridge Crystallographic Data Centre via http://www.ccdc.cam.ac.uk/structures.

ORTEP of **4** is shown below.

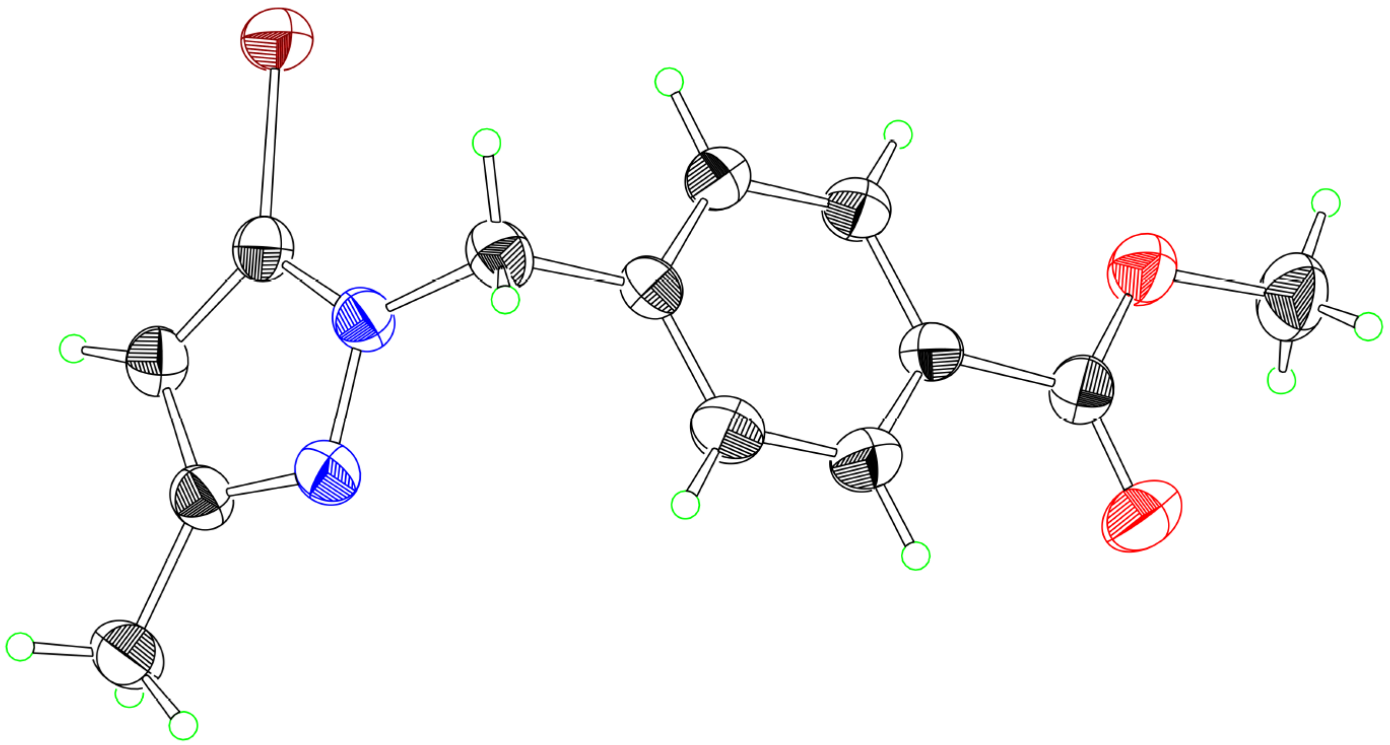

Figure S1. ORTEP of **4**, thermal ellipsoids are drawn at 30% probability.

2. HPLC chromatograms in radiosynthesis of [^{18}F]**16a**.

A: HPLC chart for purification of [^{18}F]16a

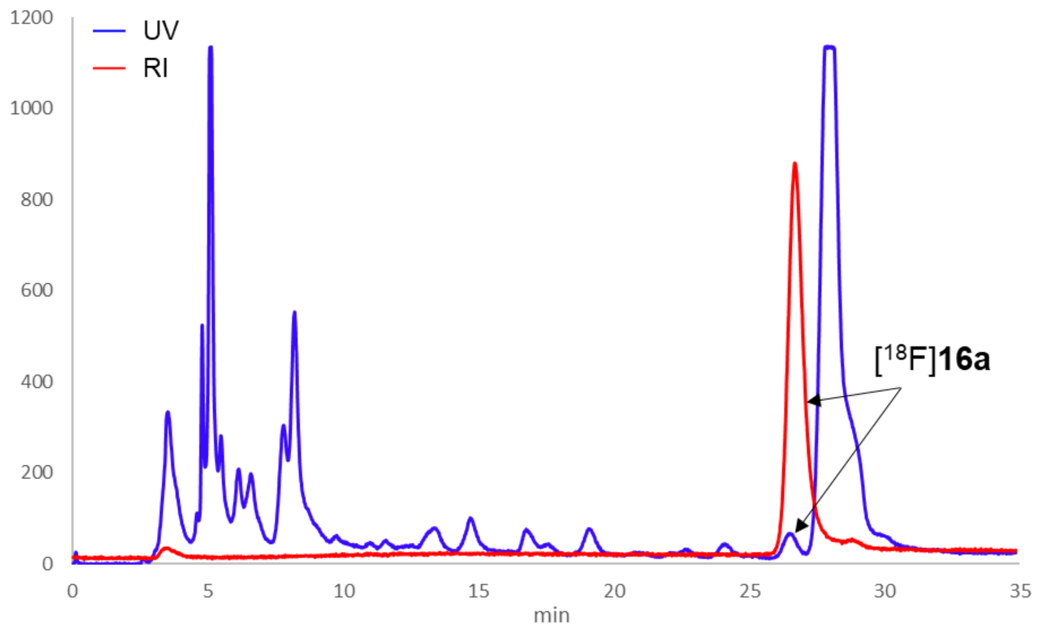

B: Analytical HPLC charts of [^{18}F]16a

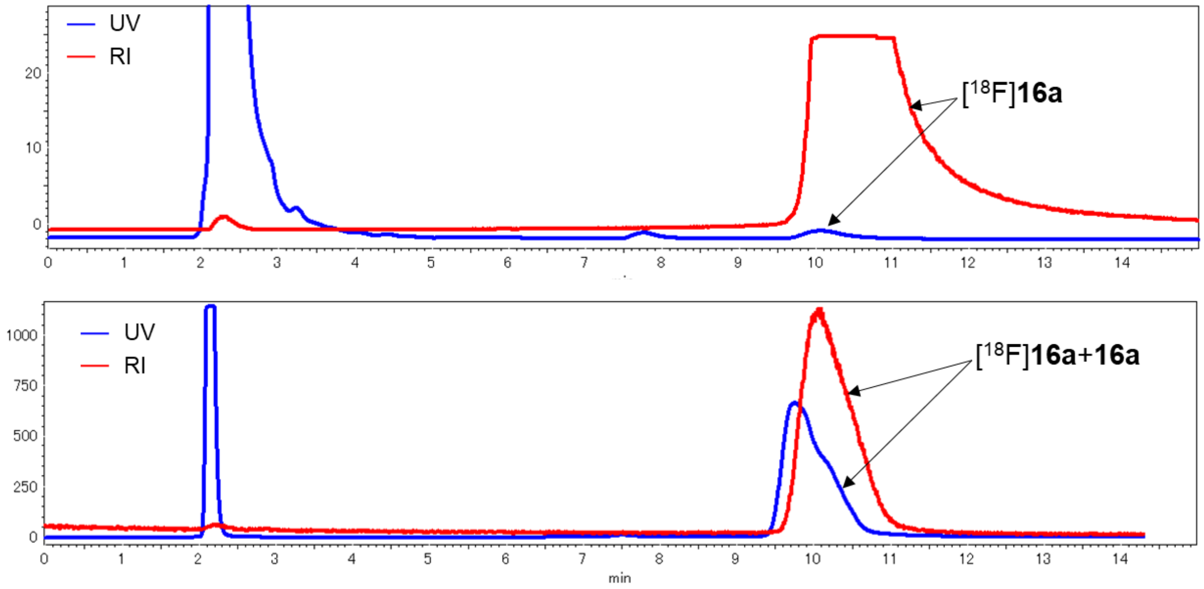

Figure S2. HPLC chromatograms in radiosynthesis of [^{18}F]**16a**.

3. Metabolite-corrected plasma input function in NHP PET imaging

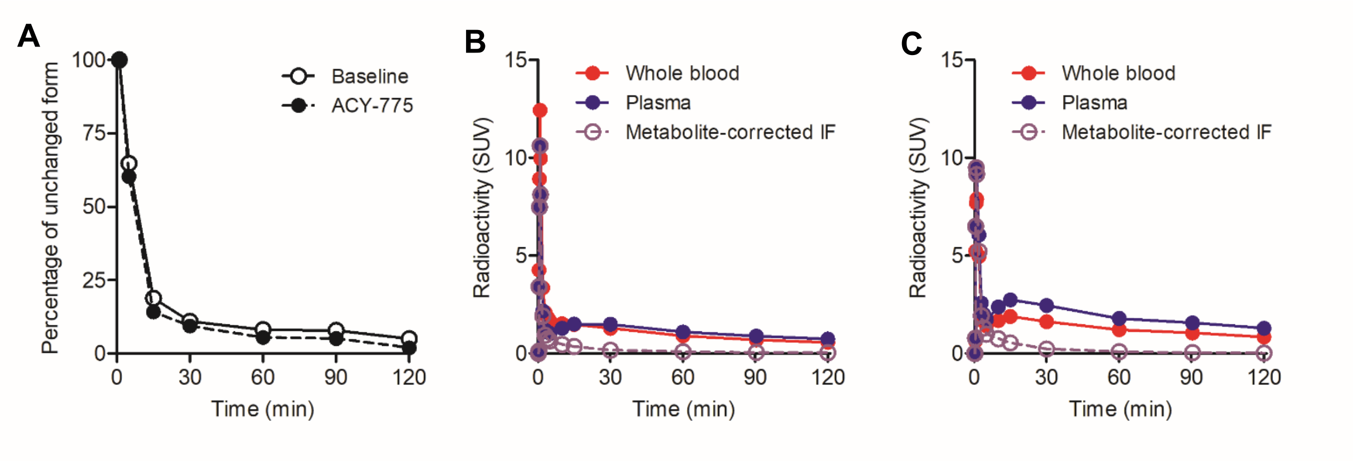

Figure S3. Percentages of unchanged form of [^{18}F]**16a** (A) in the plasma of a rhesus monkey administrated without (baseline, open circles) or with ACY-775 (2 mg/kg, buried circles) and time-course of radioactivities in the whole blood (red buried circles), plasma (blue buried circles), and metabolite-corrected input function (IF) (violet open circles) of the baseline (B) and blocking (C).

4. Representative radio-HPLC chromatograms in the plasma of baseline subject

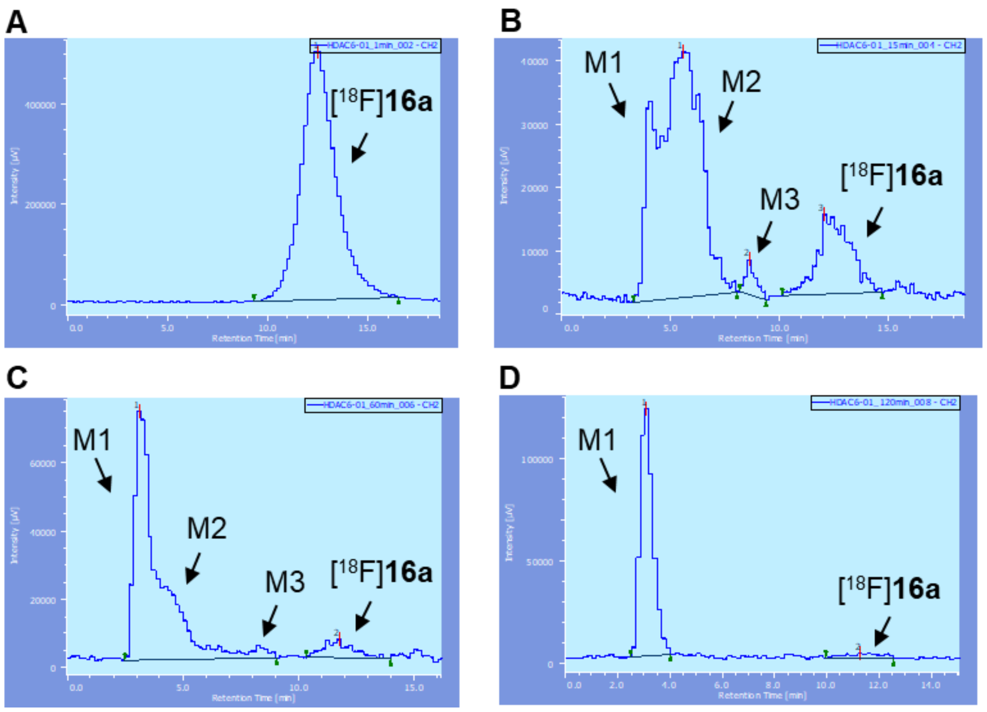

Figure S4. Representative radio-HPLC chromatograms in metabolite analysis. Chromatograms were obtained from plasma samples collected from a baseline subject at 1 min (A), 15 min (B), 60 min (C), and 120 min (D) after the injection of [^{18}F]**16a**.

5. References

(1) Burla, M. C.; Caliandro, R.; Camalli, M.; Carrozzini, B.; Cascarano, G. L.; De Caro, L.; Giacovazzo, C.; Polidori, G.; Siliqi, D.; Spagna, R. *J. Appl. Cryst.* **2007**, *40*, 609-613.

(2) Sheldrick, G.M. *Acta Cryst. A* **2008**, *64*, 112-122.
